# Inner Speech: Development, Cognitive Functions, Phenomenology, and Neurobiology

**DOI:** 10.1037/bul0000021

**Published:** 2015-05-25

**Authors:** Ben Alderson-Day, Charles Fernyhough

**Affiliations:** 1 Department of Psychology, Durham University

**Keywords:** auditory verbal hallucinations, covert speech, developmental disorders, private speech, working memory

## Abstract

Inner speech—also known as covert speech or verbal thinking—has been implicated in theories of cognitive development, speech monitoring, executive function, and psychopathology. Despite a growing body of knowledge on its phenomenology, development, and function, approaches to the scientific study of inner speech have remained diffuse and largely unintegrated. This review examines prominent theoretical approaches to inner speech and methodological challenges in its study, before reviewing current evidence on inner speech in children and adults from both typical and atypical populations. We conclude by considering prospects for an integrated cognitive science of inner speech, and present a multicomponent model of the phenomenon informed by developmental, cognitive, and psycholinguistic considerations. Despite its variability among individuals and across the life span, inner speech appears to perform significant functions in human cognition, which in some cases reflect its developmental origins and its sharing of resources with other cognitive processes.

When people reflect upon their own inner experience, they often report that it has a verbal quality (Baars, 2003). Also referred to as *verbal thinking*, *inner speaking, covert self-talk*, *internal monologue*, and *internal dialogue*, inner speech has been proposed to have an important role in the self-regulation of cognition and behavior in both childhood and adulthood, with implications for inner speech dysfunction in psychiatric conditions and developmental disorders involving atypical language skills or deficits in self-regulation (Diaz & Berk, 1992; Fernyhough, 1996; Vygotsky, 1934/1987). Despite its apparent importance for human cognition, inner speech has received relatively little attention from psychologists and cognitive neuroscientists, partly due to methodological problems involved in its study. Nevertheless, a large body of empirical work has arisen relating to inner speech, albeit in rather disparate research areas, and it plays an increasingly prominent role in psychological theorizing (Dolcos & Albarracín, 2014; Fernyhough & McCarthy-Jones, 2013; Hurlburt, Heavey, & Kelsey, 2013; Oppenheim & Dell, 2010; Williams, Bowler, & Jarrold, 2012).

The aim of the present article is to review the existing empirical work on inner speech and provide a theoretical integration of well-established and more recent research findings. First, we summarize the key theoretical positions that have been advanced relating to the development, cognitive functions, and phenomenology of inner speech. We then consider methodological issues that attend the study of inner speech. Next, we consider how inner speech emerges in childhood. In the fourth section, we consider the phenomenology of inner speech in adulthood along with its cognitive functions. We then review what is known about inner speech in atypical populations before considering neuropsychological evidence relevant to theorizing about its functional significance. Finally, we consider prospects for an integrated cognitive science of inner speech, combining developmental, cognitive, psycholinguistic, and neuropsychological evidence to provide a multicomponent model of the phenomenon.

Inner speech can be defined as the subjective experience of language in the absence of overt and audible articulation. This definition is necessarily simplistic: as the following will demonstrate, experiences of this kind vary widely in their phenomenology, their addressivity to others, their relation to the self, and their similarity to external speech. Inner speech, on these terms, incorporates but does not reduce to phenomena such as subvocal rehearsal (the use of phonological codes for the maintenance of information in working memory). The concept is also sometimes used interchangeably with *thinking*, to the extent that a close focus on the phenomenological, developmental, and cognitive features of inner speech necessitates a certain amount of redefinition of that term. In what follows, we will avoid talking about *thinking* in favour of mental processes that can be more tightly specified.

Given this diversity in terminology, our literature search covered a broad range of research areas and depended considerably on secondary sources and citation lists of key articles. *Web of Knowledge*, *PsycINFO*, and *Google Scholar* were searched for articles published from 1980–2014 containing the following keywords: *inner speech, private speech, self-talk, covert speech, silent speech, verbal thinking, verbal mediation, inner monologue, inner dialogue, inner voice, articulatory imagery, voice imagery, speech imagery,* and *auditory verbal imagery*. Both empirical and theoretical articles were permitted. Studies that only covered externalized forms of self-talk were generally not included, unless they referred to a relevant effect or population where inner speech data were not available; for instance, to our knowledge there have been no studies specifically studying inner speech in attention deficit hyperactivity disorder (ADHD), but there is research on private speech (e.g., Corkum, Humphries, Mullane, & Theriault, 2008). Where a recent review on a topic had been published (such as Hubbard, 2010, on auditory imagery; or Winsler et al., 2009, on private speech) we chose to selectively discuss studies in that area, and refer the reader to relevant summaries.

## Theories of Inner Speech

Noting a possible reason for the relative neglect of the phenomenology of inner speech, Riley (2004) observes that “the fact of its insistent indwelling can blind us to its peculiarities” (p. 8). And yet inner speech has long had an important role to play in psychological theorizing. Plato (undated 1987) noted that a dialogic conversation with the self is a familiar aspect of human experience. Although inner speech figures in a variety of psychological, neuroscientific, and philosophical discourses (Fernyhough, 2013), its nature, development, phenomenology, and functional significance have received little theoretical or empirical attention. One reason for this is that inner speech by definition cannot be directly observed, limiting the scope for its empirical study and requiring the development of methodologies for studying it indirectly (see Methodological Issues). While there exists a range of theoretical perspectives on inner speech (e.g., Larrain & Haye, 2012; Morin, 2005; Oppenheim & Dell, 2010), two in particular have proved influential for theorizing about its cognitive functions. One relates to the development of verbal mediation of cognition and behavior, and one relates to rehearsal and working memory.

### Vygotsky’s Theory

In Vygotsky’s (1934/1987) theory of cognitive development, inner speech is the outcome of a developmental process. Vygotsky assumed that understanding how such a phenomenon emerges over the life span is necessary for full comprehension of its subjective qualities and functional characteristics. Via a mechanism of internalization, linguistically mediated social exchanges (such as those between the child and a caregiver) are transformed, in Vygotsky’s model, into an internalized “conversation” with the self. The development of verbal mediation is envisaged as the process through which children become able to use language and other sign systems to regulate their own behavior. Prelinguistic intelligence is thus reshaped by language to create what Vygotsky and his student Luria termed a “functional system,” a key concept in their antimodularist view of functional localization in the brain (Fernyhough, 2010; Luria, 1965; Vygotsky, 1934/1987).

Vygotsky formulated his view of inner speech in contrast to the theory of John B. Watson. Best known as a founder of behaviorism, Watson saw inner speech (which he identified with “thinking”) as resulting from a process of the gradual reduction of self-directed speech: in other words, a purely mechanical process in which speech becomes quieter and quieter until it is first merely a whisper, and then silent thought (Watson, 1913). This view of inner speech as subvocalized language was, Vygotsky believed, mistaken (Berk, 1992). Rather, he contended, inner speech is profoundly transformed in the process of internalization, and its development involves processes more complex than the mere attenuation of the behavioral components of speaking.

Vygotsky saw support for his theory in the phenomenon now known as *private speech* (previously *egocentric speech*), in which children talk to themselves while engaged in a cognitive task. In Vygotsky’s (1934/1987) theory, private speech represents a transitional stage in the process of internalization in which interpersonal dialogues are not yet fully transformed into intrapersonal ones. Vygotsky saw private speech as having a primary role in the self-regulation of cognition and behavior, with the child gradually taking on greater strategic responsibility for activities that previously required the input of an expert other (such as a caregiver). Empirical research since Vygotsky’s time has challenged this unifunctional view of private speech, with self-directed talk now proposed to have multiple functions including pretense, practice for social encounters, language practice, and so on (Berk, 1992). Most studies point to private speech being an almost universal feature of development (Winsler, De León, Wallace, Carlton, & Willson-Quayle, 2003), although there are important individual differences in frequency and quality of self-talk (Lidstone, Meins, & Fernyhough, 2011). It is also now acknowledged that private speech does not atrophy after the completion of internalization, but can persist into adulthood as a valuable self-regulatory and motivational tool.

As noted, the developmental transition envisaged by Vygotsky (from social to private to inner speech) was proposed to be accompanied by both syntactic and semantic transformations (see Fernyhough & McCarthy-Jones, 2013). Internalization involves the abbreviation of the syntax of internalized language, which results in inner speech having a “note-form” quality (in which the “psychological subject” or topic of the utterance is already known to the thinker) compared with external speech. Vygotsky identified three main semantic transformations accompanying internalization: the *predominance of sense over meaning* (in which personal, private meanings achieve a greater prominence than conventional, public ones); the process of *agglutination* (the development of hybrid words signifying complex concepts); and the *infusion of sense* (in which specific elements of inner language become infused with more semantic associations that are present in their conventional meanings). For example, a word like “interview” might have a clear referent (an upcoming appointment), but its sense could mean much more when uttered in inner speech: worry, performance anxiety, hopes for the future, or the need to prepare.

Vygotsky’s ideas about inner speech have been extended in recent theoretical and empirical research. Fernyhough (2004) proposed that inner speech should take two distinct forms: *expanded inner speech*, in which internal dialogue retains many of the phonological properties and turn-taking qualities of external dialogue, and *condensed inner speech*, in which the semantic and syntactic transformations that accompany internalization are taken to their conclusion, and inner speech approaches the state of “thinking in pure meanings” described by Vygotsky (1934/1987). In this latter form of inner speech, the phonological qualities of the internalized speech are attenuated and the multiple perspectives (Fernyhough, 1996, 2009a) that constitute the dialogue are manifested simultaneously. In Fernyhough’s model, the default setting for inner speech is condensed, with the transition to expanded inner speech resulting from stress and cognitive challenge.

Recent empirical research has been largely supportive of Vygotskian claims about the functional significance of private speech, particularly its relations to task difficulty and task performance (Al-Namlah, Fernyhough, & Meins, 2006; Fernyhough & Fradley, 2005; Winsler, Fernyhough, & Montero, 2009), and its developmental trajectory (Winsler & Naglieri, 2003). Vygotsky’s ideas about the role of such mediation in self-regulation have begun to be integrated into modern research into the executive functions, the heterogeneous set of cognitive capacities responsible for the planning, inhibition, and control of behavior (e.g., Cragg & Nation, 2010; Williams, Bowler, & Jarrold, 2012). One implication of Vygotsky’s theory, that inner speech is dialogic in nature, has been proposed to be important in domains such as social understanding (Davis, Meins, & Fernyhough, 2013) and creativity (Fernyhough, 2008, 2009a). Inner speech has also been proposed to have an important role in metacognition, self-awareness, and self-understanding (Morin, 2005).

### Inner Speech in Working Memory

A second important theoretical perspective concerns the role of inner speech in working memory. Working memory refers to the retention of information “online” during a complex task, such as keeping a set of directions in mind while navigating around a new building, or rehearsing a shopping list.

Models of working memory vary in terms of whether it is considered a single or multicomponent process, its relation to attention, and the importance of individual differences (Miyake & Shah, 1999). The theory most pertinent to discussing inner speech—and still the most influential approach—is that derived from Baddeley and Hitch’s (1974) multicomponent model. Baddeley and Hitch proposed that working memory comprised three components: a central executive, responsible for the allocation and management of attentional resources; the phonological (sometimes known as the articulatory) loop, a slave system responsible for the representation of acoustic, verbal, or phonological information; and a visuospatial scratchpad, a slave system that serves visual and spatial aspects of task-based short-term memory (STM). Baddeley (2000) also added a fourth component, the episodic buffer, a multimodal temporary store that can bind concurrent stimuli and draw on information from long-term memory.

The distinction between slave systems in Baddeley and Hitch’s model has produced a large body of research on the operations of verbal working memory. In this model, the phonological loop is made up of two subcomponents: a passive, phonological store, with a decay time of 1–2 s, and an active rehearsal mechanism that uses offline speech planning processes—in other words, inner speech, or something very similar (Baddeley, 1992).

Support for the independence of a phonological loop from other working memory processes has largely come from evidence of interference effects in dual-task studies. In such paradigms, participants are asked to encode a set of target stimuli—such as learning a list of words—while engaging in a secondary task which either involves verbal or visuospatial processing. A typical verbal distractor method is articulatory suppression: engaging the articulators in a separate task (such as repeating days of the week) has been shown to disrupt memory for verbal material in numerous studies (e.g., Baddeley, Lewis & Vallar, 1984). In contrast, tapping out particular spatial patterns selectively affects visuospatial working memory skills, leading to impaired recall in that domain only (Logie, Zucco, & Baddeley, 1990).

Evidence of verbal representations in the memory trace comes from common memory effects related to specific verbal and phonological properties. For instance, words that take longer to say overtly reduce overall recall, suggesting a “word length effect” on the memory trace (Baddeley, Thomson, & Buchanan, 1975). Words that sound the same are also prone to confusion, leading to poorer recall for the whole list of items: this “phonological similarity effect” influences maintenance of verbal material, but also visual material that has been verbally rehearsed (Conrad & Hull, 1964).

Developmentally, there is evidence that the different components of working memory follow different trajectories of maturation, and that this divergence of developmental pathways begins relatively early (Alloway, Gathercole, & Pickering, 2006). Although the evidence is not unequivocal, it is generally agreed that children begin to use verbal mediation of STM from around 7 years of age (Gathercole, 1998), at which point they begin to be susceptible to the phonological similarity effect (Conrad, 1971; Gathercole & Hitch, 1993) and word length effect (Baddeley, Thomson, & Buchanan, 1975). The ability to hold phonological representations in mind, however, appears to come online much earlier, possibly as young as 18 months (e.g., Mani & Plunkett, 2010). One way of interpreting this evidence is to think that the phonological loop primarily functions as a language-learning tool, as evidenced in its use in the first phases of language acquisition in infancy (Baddeley, Gathercole, & Papagno, 1998).

### Comparing Vygotskian and Working Memory Approaches to Inner Speech

To date, there have been few attempts to integrate the Vygotskian and working memory approaches to inner speech (although see Al-Namlah et al., 2006). One objection that is occasionally raised regarding integration of Vygotskian and working memory accounts is that, because an operational phonological loop predates the emergence of private speech, inner speech development cannot be driven by private speech internalization (Hitch, Halliday, Schaafstal, & Heffernan, 1991; Perrone-Bertolotti, Rapin, Lachaux, Baciu, & Lœvenbruck, 2014). The presence of a phonological loop indeed rules out the suggestion that an earlier stage of private speech is *necessary* for the development of verbal mentation. However, as Al-Namlah, Fernyhough, and Meins (2006) point out, this objection misunderstands the Vygotskian position, which prioritizes the question of how language is employed in internal self-regulation above the neural or cognitive substrates that make language use possible. Put another way, the working memory approach largely confines itself to questions of what inner speech is necessary for (i.e., verbal rehearsal and recoding), whereas a Vygotskian approach describes the contingent use of inner speech as a tool for enhancing and transforming other developing cognitive functions.

## Methodological Issues

As a psychological process with no overt behavioral manifestation, inner speech has traditionally been considered difficult or impossible to study empirically. However, recent methodological advances have meant that a range of direct and indirect methods exist for studying inner speech. Some methods have been designed to encourage inner speech and examine its effects; some have sought to block or inhibit inner speech and observe which other processes are also impacted. Finally, some techniques have sought to “capture” inner speech processes spontaneously, during the course of everyday life.

### Questionnaires

The simplest approach to investigating inner speech is to ask people to report directly on its occurrence. Such methods are particularly valuable for investigating inner speech frequency, context dependence, and phenomenological properties, although their veridicality has often been questioned (for a recent example see Hurlburt et al., 2013).

Questionnaire approaches to inner speech tend to follow typical steps for scale development. For example, McCarthy-Jones and Fernyhough (2011) generated statements about the quality and structure of inner speech and submitted them to exploratory and confirmatory factor analysis in two undergraduate samples, resulting in an 18-item Varieties of Inner Speech Questionnaire (VISQ). Other self-report scales assess features such as inner speech frequency, content, and context (e.g., Duncan & Cheyne, 1999). Although such scales often report acceptable psychometric reliability, correlations among scales can be weak (Uttl, Morin, & Hamper, 2011), indicating limited validity, or that scales are measuring different aspects of a complex, multifaceted construct.

### Experience Sampling

While questionnaires are typically used to ask about inner speech in general or across a particular time period, experience sampling methods (Csikszentmihalyi & Larson, 1987) aim for momentary assessments of inner speech, selected at random. The virtue of such approaches is that they avoid the need for participants to make a general judgment about the extent and nature of their inner speech, usually asking only about the contents of experience at the moment of a random alert (such as a beep).

Some experience sampling techniques will use the same or similar items as questionnaires that ask about inner speech; others have used diary or thought-listing techniques to prompt participants to report on their experience in a more open-ended way (e.g., D’Argembeau et al., 2011). Other researchers prefer to use detailed introspective interviews as part of their experience sampling approach. Considered methodologically problematic for a long time due to the impossibility of objective verification, introspective methods have undergone a resurgence of interest in recent years (Hurlburt & Schwitzgebel, 2007). One highly developed method, Descriptive Experience Sampling (DES), involves training participants to report on their own inner experience in the moment before a random alert, first through making brief notes for themselves and then through a detailed expositional interview. As will be discussed, using DES to assess inner speech reveals striking phenomenological richness and diversity, which in some cases appears to contradict findings from self-report questionnaires (Hurlburt et al., 2013). However, the extensive and iterative interview processes involved in DES have also been questioned for the extent to which they may shape and change the experiences that participants report (see Hurlburt & Schwitzgebel, 2007).

### Private Speech as an Indicator of Inner Speech

One indirect approach to researching inner speech is through the study of what Vygotsky held to be its observable counterpart, private speech. For example, Al-Namlah et al. (2006) investigated whether Vygotsky’s ideas about the development of verbal mediation in childhood would be evidenced in a domain-general transition to verbal self-regulation. They found that use of self-regulatory private speech on a “Tower of London” task (a commonly used measure of planning, where participants must move rings on a set of poles to match a particular arrangement) correlated with the size of the phonological similarity effect, an index of inner speech use in working memory. Such a finding suggests close links between private speech and covert verbal encoding.

There are difficulties, however, with taking private speech as a direct proxy for inner speech: for instance, extensive private speech use in some children could reflect a lack of internalized inner speech, while an outwardly silent child could be using inner speech all the time. Subtle signs of inner speech can also be coded alongside private speech. For example, Fernyhough and Fradley (2005) used a coding frame (based on Berk, 1986) that distinguished between social speech (vocalizations during a task that were clearly addressed to someone), private speech (nonaddressed overt vocalizations), and task-relevant external manifestations of inner speech (indecipherable lip and tongue movements or silent articulatory behavior during a task).

### Dual-Task Methods

Another indirect methodology that escapes some of these concerns is the use of dual-task designs. The rationale here is that interfering with or blocking inner speech, through a secondary task that prevents subvocal articulation, can be investigated in relation to deficits on a primary task (similarly to how such methods are used in working memory studies). Articulatory suppression to interfere with inner speech on cognitive tasks has been widely used in children and adults (Baldo et al., 2005; Hermer-Vazquez, Spelke, & Katsnelson, 1999; Lidstone, Meins, & Fernyhough, 2010). Ideally articulatory suppression is deployed along with an additional condition including a nonverbal task, such as spatial tapping, as this allows investigators to control for general effects of dual-tasking and to identify effects specific to inner speech processes. In working memory studies, a further control task is sometimes included to interfere with the central executive: random number generation, for example, is thought to block both articulatory/phonological slave processes and to require direct attention from the central executive in order to avoid generating nonrandom number sequences (Baddeley, 1996).

### Phonological Judgments

An alternative method of studying inner speech, which overlaps with methods used in auditory imagery research, is to ask participants to make judgments based on the contents of their inner speech. For example, participants may be required to judge whether given words or sentences rhyme, or count the syllables in a given word (Filik & Barber, 2011; Geva, Bennett, Warburton, & Patterson, 2011). Such methods have been argued to provide a more objective test of inner speech use than self-report methods (Hubbard, 2010). However, it should be noted that judgment tasks of this kind often assume that phonological properties of inner speech are in some way being consulted, rather than the decision being based on other available stimulus information (rhyming judgments, for instance, could also be based on orthographic features of word stimuli).

### Neuroimaging and Neuropsychology

Finally, a number of studies have either used functional neuroimaging techniques or neuropsychological case studies to examine the neural substrates of inner speech. Such studies have been conducted since the earliest days of neuroimaging (McGuire et al., 1995), and have been driven primarily by an interest in the possible role of inner speech in the experience of auditory verbal hallucinations (see Adult Psychopathology), although neuroimaging research on verbal working memory (e.g., Marvel & Desmond, 2010) and imagery for speech (Tian & Poeppel, 2010) has also made an important contribution.

Typical inner speech elicitation methods include subvocal articulation of words and sentences or imagining speech with varying characteristics (e.g., first- vs. third-person speech, or fast vs. slow speech; Shergill et al., 2002). Such studies have been criticized for their lack of ecological validity in eliciting inner speech, and for their failure to recognise the possibility of inner speech continuing during baseline assessments (Jones & Fernyhough, 2007). Furthermore, some elicitation paradigms for inner speech have not adopted behavioral controls to check whether inner speech is actually produced during scanning experiments, relying instead on participants’ self-reported acquiescence with the task (this problem is also faced in auditory imagery research; see Zatorre & Halpern, 2005, for a discussion). Approaches for counteracting this include the administration of behavioral tasks that require internal phonological judgments: asking participants to judge the metric stress of simple words, for example, is thought to require internal inspection of speech (Aleman et al., 2005). Neuroimaging findings relating to inner speech are considered in Inner Speech in the Brain.

## Development of Inner Speech

Studying the development of inner speech can give us important information about its phenomenological qualities and psychological functions. Researching inner speech in childhood presents specific methodological challenges, including participants’ compliance with dual-task demands (e.g., articulatory suppression), limitations on the richness of child participants’ experience sampling reports, and age-related restrictions on neuroimaging.

### Private Speech as a Precursor of Inner Speech

The methodological challenges that attend the study of inner speech have led to a focus on its observable developmental precursor, private speech, as a window onto its development. Key questions that have been examined include the emergence and apparent extinction of private speech, the social context within which self-directed speech is observed, and the role of verbal mediation in supporting specific activities. Much of the prior literature on private speech was outlined in an extensive review by Winsler (2009); accordingly, this section provides a brief overview of private speech findings in children, with reference to some more recent studies.

As noted above, private speech is an almost universal feature of young children’s development. It was first described by Piaget in the 1920s, who considered it as evidence of young children’s inability to adapt their communications to a listener (hence, his term *egocentric speech*). Private speech has subsequently been shown to have a significant functional role in the self-regulation of cognition and behavior. Typically emerging with the development of expressive language skills around age 2–3, private speech frequently takes the form of an accompaniment to or commentary on an ongoing activity. A regular occurrence between the ages of 3 and 8, private speech appears to follow a trajectory from overt task-irrelevant speech, to overt task-relevant speech (e.g., self-guiding comments spoken out loud), to external manifestations of inner speech (e.g., whispering, inaudible muttering; Berk, 1986; Winsler, Diaz, & Montero, 1997).

In line with Vygotsky’s theory, the occurrence of self-regulatory private speech is associated in some studies with task performance and task difficulty (e.g., Fernyhough & Fradley, 2005), and demonstrates some of the structural changes, such as abbreviation, hypothesized to attend internalization (Goudena, 1992). There is evidence to support Vygotsky’s claim that self-regulatory speech “goes underground” in middle childhood to form inner speech, with private speech peaking in incidence around age 5 (Kohlberg, Yaeger, & Hjertholm, 1968) and then declining in parallel with a growth in inner speech use (Damianova, Lucas, & Sullivan, 2012) as defined by Fernyhough and Fradley’s (2005) criteria. However, there is also evidence for continuing high levels of private speech use well into the elementary school years (Berk & Garvin, 1984; Berk & Potts, 1991) and indeed into adulthood (Duncan & Cheyne, 2001; Duncan & Tarulli, 2009). Examples of continued use of private speech, however, do not necessarily indicate similar functions or benefits for performance: comparing verbal strategy use on cognitive tasks in children aged 5–17, Winsler and Naglieri (2003) showed that 5-year-olds but not older children performed better on tasks when they used more overt speech, even though private speech persisted well beyond this age.

Despite its proposed origins in social interaction (Furrow, 1992; Goudena, 1987), social influences on private speech have not been studied extensively in recent years. In one recent exception, McGonigle-Chalmers, Slater, and Smith (2014) studied the extent to which private speech use is moderated by the presence of another person in the room when 3- to 4-year-old children attempted a novel sorting task. Out-loud commentaries—which typically narrated or explained what was happening during the task—were significantly more prevalent when another person was in the room, suggesting a social, declarative function of private speech. Ratings were also made of incomplete or mumbled speech commentaries, which were suggestive of inner speech being used during the task, but notably these did not change significantly with the presence or absence of another person. Thus, the production of overt private speech may be socially sensitive while inner speech or more covert processes retain a necessarily private and self-directed role.

These findings are in line with Vygotsky’s original observations that private speech depends on children’s understanding that they are in the presence of an interlocutor who can understand them, and are consistent with his view that private speech emerges through a differentiation of the social regulatory function of social speech, with speech that was previously used to regulate the behavior of others gradually becoming directed back at the self. They are also congruent with Piaget’s (1959) interpretation of private speech as representing a failed attempt to communicate, and with Kohlberg, Yaeger, and Hjertholm’s (1968) characterization of private speech as a “parasocial” phenomenon.

The social relevance of private speech is also supported by recent research on imaginary companions in childhood. Davis, Meins, and Fernyhough (2013) studied private speech during free play and imaginary companion (IC) status in a large sample of 5-year-olds (*n* = 148). Children with an IC used significantly more covert private speech during free play than those without an IC, a relation that was evident even when controlling for effects of socioeconomic status, receptive language skill, and total number of utterances. Although a causal direction cannot be specified, these findings suggest that individual differences in creative and imaginative capacities are important to consider in gauging the developmental role of private speech.

Thus, while Vygotsky’s model of the developmental significance of self-directed speech has been well supported by empirical research, private speech may have functions that go beyond self-regulation of cognition and behavior. Private speech appears to have a role in emotional expression and regulation (Atencio & Montero, 2009; Day & Smith, 2013), planning for communicative interaction (San Martin, Montero, Navarro, & Biglia, 2014), theory of mind (Fernyhough & Meins, 2009), self-discrimination (Fernyhough & Russell, 1997), fantasy (Olszewski, 1987), and creativity (White & Daugherty, 2009). Engaging in private speech has also recently been proposed to have a role in the mediation of children’s autobiographical memory (Al-Namlah, Meins, & Fernyhough, 2012). It seems likely that private speech is a multifunctional phenomenon; comparisons with the functionality of its putative counterpart, inner speech, are considered below.

### The Cognitive Functions of Inner Speech in Childhood

Children’s adoption of inner speech is evidenced relatively early in development in the apparent emergence of the phonological similarity effect around age 7 (Gathercole, 1998). The effect is typically evidenced when visually presented items that are phonologically similar prove harder to recall than phonologically dissimilar items, due to interference between item words that sound the same. When children are asked to learn a set of pictures, those aged 7 and over tend to exhibit a phonological similarity effect, suggesting that visual material is being recoded into a verbal form via subvocal rehearsal (i.e., inner speech). Children younger than 7, in contrast, tend not to demonstrate this effect, suggesting an absence of verbal rehearsal strategies (Henry, Messer, Luger-Klein, & Crane, 2012).

This conclusion has recently been questioned by Jarrold and Citroen (2013) who argue that the apparent emergence of the phonological similarity effect at age 7 does not necessarily reflect a qualitative change in strategy. In a study of 5- to 9-year-old children, they tested recall for verbally versus visually presented items, while also varying the mode of response (verbal or visual reporting), to examine whether verbal recoding of visually presented items specifically showed a change with age. While visual encoding plus verbal reporting demonstrated the most prominent phonological similarity effect, interactions between age and similarity were evident in each condition; that is, even when verbally recoded rehearsal was not specifically required. In addition, a simulation model indicated that the lack of an effect in younger children could be explained by floor effects in recall for other, dissimilar items to be remembered. Thus, evidence of phonological similarity effects may emerge around age 7 not because of an adoption of rehearsal strategies at this time, but as a result of gradual changes in overall recall skill.

Jarrold and Citroen’s finding does not undermine the idea that children may generally tend to utilize verbal rehearsal more with age, but suggests that the presence or absence of a phonological similarity effect should not be taken to indicate a specific, qualitative shift in children’s inner speech strategies (see also Jarrold & Tam, 2010). Moreover, it highlights the need (also considered by Al-Namlah et al., 2006) to evaluate children’s use of verbal strategies in the context of their other skills, such as STM capacity.

Whether or not children’s use of inner speech undergoes a qualitative change in early to middle childhood, there is good evidence to suggest that it plays an increasingly prominent role in supporting cognitive operations in this developmental period. Most of the work in this area has concerned the role of verbal strategies in supporting complex executive functions such as cognitive flexibility and planning. Concerning the former, the ability to represent linguistic rules to guide and support flexible behavior has been proposed as a core part of executive functioning development during childhood (Zelazo, Craik, & Booth, 2004; Zelazo et al., 2003). In general, younger children (3- to 5-year-olds) will struggle with tasks requiring a switch between two different response rules, whereas older children will not. Evidence to suggest that this involves verbal processes is provided by reductions in performance on such tasks under articulatory suppression (e.g., Fatzer & Roebers, 2012) and improvements in performance when participants are encouraged to use verbal cues (Kray, Gaspard, Karbach, & Blaye, 2013). Younger but not older children appear to benefit from the prompt to use verbal labels, both on switching tasks (Kray, Eber, & Karbach, 2008) and in other contexts (see Müller, Jacques, Brocki, & Zelazo, 2009, for a review), suggesting a lack of spontaneous inner speech use at younger ages.

What exactly inner speech is doing to support performance in this way is not always clear: in a review of child and adult switching studies, Cragg and Nation (2010) noted that verbalized strategies speed up performance on switch and nonswitch trials but do not necessarily facilitate the act of switching itself. If so, this would suggest that inner speech is helping to maintain a specific response set, or is acting as a reminder of task and response order, rather than being involved in flexible responding *per se*. In any case, use of inner speech appears to become a key strategy in switching tasks during childhood, and there is evidence of this strategic use continuing into adulthood (e.g., Emerson & Miyake, 2003, see Cognitive Functions of Inner Speech in Adulthood).

Research on planning and verbal strategies in childhood has almost exclusively been conducted using tower tasks, such as the Tower of London task (Shallice, 1982) or the very similar Tower of Hanoi puzzle. As noted previously, tower tasks require participants to move a set of rings or disks from one arrangement to another across three columns. Although fundamentally a visuospatial problem, the number of possible moves to a solution creates a problem-space bigger than visuospatial working memory capacity will typically allow, meaning that verbal strategies often come into play.

Private speech on such tasks has been observed to increase in relation to task difficulty in children (Fernyhough & Fradley, 2005) and correlates with other indicators of verbal strategy use, such as susceptibility to the phonological similarity effect on STM tasks (Al-Namlah et al., 2006). Concerning inner speech specifically, Lidstone, Meins, and Fernyhough (2010) compared Tower of London performance in children under articulatory suppression, foot-tapping, and normal conditions. Performance (as indicated by percentage of correct trials) was selectively impaired during articulatory suppression, and the size of the performance decrement correlated with private speech use in the control condition, although this was only evident when participants were specifically instructed to plan ahead. Effects of articulatory suppression on Tower of London performance have also been reported in the control groups of typically developing children in studies on autism (e.g., Wallace, Silvers, Martin, & Kenworthy, 2009), but these effects have not always been clearly separable from other dual-task demands (Holland & Low, 2010).

The apparent use of verbal strategies in recall, switching, and planning tasks, and correlations among them (e.g., Al-Namlah et al., 2006), are suggestive of a domain-general shift to verbal mediation in early childhood, affecting processes as different as STM and problem-solving. However, it seems likely that inner speech use across domains may still follow separable trajectories and be guided by the specific demands of each task. The data from studies of cognitive flexibility and other executive domains suggest that, even within a given task, inner speech will only be a useful strategy in particular conditions: naming stimuli, for example, appears to speed up response execution, but naming the response required (e.g., *stop* or *go*) does not (Kray, Kipp, & Karbach, 2009). There is also still a relative lack of research comparing strategy use across multiple domains. In one recent exception, Fatzer and Roebers (2012) observed strong effects of articulatory suppression on complex memory span (i.e., working memory), medium effects on a measure of cognitive flexibility, and little effect on a test of selective attention. If these processes are taken to follow separate rates of maturation, it seems likely be that inner speech offers a domain-general tool that is only selectively deployed when it is most relevant and beneficial to the executive functioning process at hand.

### How do Children Experience Inner Speech?

Asking people to reflect on the subjective qualities of their inner experience is fraught with difficulties, and the challenges are arguably more acute when working with children. Some attempts have been made to use experience sampling methods with children, although they have not to date focused on inner speech. For example, Hurlburt (Hurlburt & Schwitzgebel, 2007, p. 111, Box 5.8) used DES with a 9-year-old boy, who noted that the construction of a mental image (of a hole in his backyard containing some toys) took a considerable amount of time to complete. Complex or multipart images are known to take longer to generate than simple images (Hubbard & Stoeckig, 1988; Kosslyn et al., 1983), and this may particularly be the case for visual imagery in children. If this were to apply also to inner speech, it suggests that the phenomenology of verbal thinking in children may lack a certain richness and complexity. In a series of studies, Flavell and colleagues (e.g., Flavell, Flavell, & Green, 2001; Flavell, Green, & Flavell, 1993) also found limited understanding of inner experience (such as of the ongoing stream of consciousness assumed to characterize many people’s experience) in preschool children. This can be interpreted either in terms of young children’s weak introspective abilities (Flavell et al., 1993) or in terms of young children lacking adult-like inner speech, as a result of the time it takes to become internalized (Fernyhough, 2009b).

Children’s reluctance to report on inner speech, coupled with their apparent lack of awareness of it, should not necessarily be taken as indicating that they do not experience it in any form. The suggestion of links between private speech and various imaginative and creative activities, such as engaging with an imaginary companion (Davis et al., 2013), also raises the interesting question of whether inner speech plays a similar role in the inner experience of young children. The development of better methods to investigate inner speech phenomenology in children is needed to begin to answer this and related questions.

## Inner Speech in Adult Cognition

### Cognitive Functions of Inner Speech in Adulthood

Inner speech in adulthood has largely been studied as a cognitive tool supporting memory and other complex cognitive processes (see Sokolov, 1975, for an early review). Although inner speech has frequently been claimed to be important for problem-solving across different contexts, a precise account of its cognitive functions requires examination of its deployment in different task domains.

As in research with children, studies on inner speech function in adulthood have largely focused on its role in verbal STM and executive function. The use of inner speech as a rehearsal tool in working memory is perhaps its most well-known function: verbal rehearsal can refresh the memory trace continuously, provided articulation is not suppressed, and this will reliably lead to better recall (Baddeley, 1992). Even if articulation is blocked, there is evidence that the phonological store—or “inner ear”—can still maintain some phonological information, albeit in a state where it is more liable to interference and decay (Baddeley & Larsen, 2007; Smith, Reisberg, & Wilson, 1992). Articulatory suppression also removes the word-length and phonological similarity effects typically observed for verbal rehearsal (Baddeley, 2012). Contemporary research on verbal working memory in adults is extensive and will not be discussed here (see, e.g., Camos, Mora, & Barrouillet, 2013; Macnamara, Moore, & Conway, 2011). Regarding executive functions, most research has again focused on cognitive flexibility (via sorting/switching tasks) and planning (via tower tasks).

In adults, inner speech continues to be implicated in tasks that require switching between different responses and rules (Baddeley, Chincotta, & Adlam, 2001), as it does in children (Cragg & Nation, 2010). For example, Emerson and Miyake (2003) compared switching performance across a range of experiments using articulatory suppression and a foot-tapping control. The deployment of articulatory suppression consistently disrupted performance by increasing the “switch cost” between trials requiring different arithmetic rules, suggesting that inner speech acted as a tool to prepare and smooth transitions between trials. In addition, this effect was specifically moderated by the types of task cues deployed: task conditions with explicit cues reduced the effect of articulatory suppression, suggesting that inner speech was not required when the task materials sufficiently supported the required mode of response. Task difficulty, in contrast, made no difference to the articulatory suppression effect. These results suggest that inner speech facilitated performance by specifically acting as a mnemonic cue for how to respond, when such cues were lacking in the task itself.

Supporting evidence for the relevance of cue types to inner speech was provided in a follow-up experiment by Miyake, Emerson, Padilla, and Ahn (2004). Comparing switch costs for judgment tasks with full word (e.g., SHAPE) or single letter (S . . .) cues, articulatory suppression increased the switch costs for the latter but not the former. This was interpreted by Miyake et al. as evidence that inner speech was required for switching where it played a non-negligible role in the retrieval of relevant information. That is, blocking inner speech only really mattered when inner speech was needed to “fill out” the cues in the task; more explicit cues such as a full word did not recruit inner speech to the same degree, and thus no switch cost was induced. This filling out of a response is in some ways analogous to effects that have been observed for auditory imagery, where participants can have vivid and sometimes involuntary auditory experiences in the gaps of familiar songs or other sounds (e.g., Kraemer, Macrae, Green, & Kelley, 2005). Taken together, these studies suggest that inner speech has a beneficial effect on performance (by minimizing costs associated with switching), but only in conditions where verbalization seems to somehow complete the information set needed for an efficient and consistent response.

For planning, in contrast, there is perhaps less evidence for inner speech having a central role in adult task performance. While Williams et al. (2012) reported an increase in the number of moves used by adults attempting a tower task under articulatory suppression, Phillips, Wynn, Gilhooly, Della Sala, and Logie (1999) previously found no effect of interfering with inner speech on planning skills. An individual differences analysis by the latter group indicated that tower performance was closely related to visuospatial rather than verbal working memory skills (Gilhooly, Wynn, Phillips, Logie, & Della Sala, 2002; see also Cheetham, Rahm, Kaller, & Unterrainer, 2012). Similarly, in a virtual-reality study of multitasking that included the requirement to adjust complex plans midway through a task, Law et al. (2013) reported no effect of articulatory suppression on adult performance, but effects of random number generation (posited to block general executive resources) and concurrent auditory localization (requiring spatial working memory).

Inconsistencies in the planning literature imply that, while children may deploy private and inner speech during common planning tasks, adults appear to rely less on these strategies. What is important to bear in mind with such tasks, though, is that they largely require planning within a visuospatial domain. Tower tasks *can* be planned verbally, but task execution and the representation of possible states is still fundamentally a visuospatial activity. That is, it is not clear that the creation and implementation of verbal plans would be the optimal strategy on such tasks, even if children and adults spontaneously self-talk when they attempt them. Similarly, standard multitasking tasks (e.g., Law et al., 2013) often require navigation around a spatial array or environment: verbal processes may help to set up a plan, but are arguably unlikely to take priority over visuospatial skills during the commission of a plan. The contrast between child and adult deployment of self-directed speech could reflect the relative weakness of visuospatial working memory in the former (Gathercole, Pickering, Ambridge, & Wearing, 2004; Pickering, 2001), leading to compensatory use of verbal strategies to “bootstrap” performance.

Another skill closely related to planning is logical or propositional reasoning. A *prima facie* assumption may be that, if inner speech plays an integral role in certain higher cognitive processes, it would be most likely to support explicitly verbal forms of inference, such as reasoning about verbal propositions or syllogisms. Evidence to support this proposition, however, is mixed. Verbal working memory appears to be important for maintaining information about logical premises, particularly when information is encountered sequentially, but generally verbal interference does not impair this kind of reasoning any more than visuospatial forms of interference, such as spatial tapping (Gilhooly, 2005). There may be individual differences in strategy use during reasoning, with participants varying in the extent to which they report predominantly verbal or visual strategies. These individual differences appear to relate to variation in verbal and spatial working memory skills, but do not necessarily translate into differences in reasoning skill (Bacon, Handley, & Newstead, 2005). Similarly, matrix reasoning tasks, which predominantly consist of visuospatial stimuli but which can be solved using various visual or verbal strategies, do not appear to be specifically affected by articulatory suppression: for instance, Rao and Baddeley (2013) compared effects of number repetition (articulatory suppression) and backward counting (central executive interference) on matrix reasoning, and found that only the latter negatively affected the time it took to reach a solution. Thus, inner speech does not appear necessary for tasks involving logical reasoning, even for verbal material.

Beyond its putative roles in task-switching, planning, and logical reasoning, inner speech has been hypothesized to be involved in a range of other processes, including reasoning about others, spatial orientation, categorization, cognitive control, and reading. Two studies have used verbal shadowing (the immediate repetition of verbal material, postulated to block subvocal articulation) to investigate the role of language in false-belief reasoning. Newton and de Villiers (2007) compared verbal shadowing and rhythmic tapping effects on nonverbal reasoning in a sample of adults. Success rates were significantly lower for false-belief reasoning during verbal interference, but not spatial interference. In contrast, judgments about true belief were accurate across all conditions, demonstrating the specificity of the verbal effect to false-belief attribution. A more recent study by Forgeot d′Arc and Ramus (2011) also observed an interference effect of verbal shadowing, but this was not specific to false-belief reasoning; shadowing also affected reasoning about other mental states (such as agents’ goals) and mechanistic reasoning.

Employing similar techniques, Hermer-Vazquez et al. (1999) showed that verbal shadowing interfered with performance on a task requiring integration of geometric and color information, suggesting a role for inner speech in the labeling and binding of information across modalities. Using a verbal distractor task (number repetition), Lupyan (2009) reported specific effects of verbal interference on categorization skills in adults when they were asked to classify pictures based on a single perceptual dimension (e.g., color) while ignoring other relevant dimensions (such as shape). Finally, Tullett and Inzlicht (2010) compared adult response inhibition skills on a Go-NoGo task under articulatory suppression, spatial tapping, and control (single-task) conditions. Compared with spatial tapping, articulatory suppression was associated with a greater number of commission errors, an effect that was particularly exacerbated when a switching component was added to the inhibition task.

Inner speech also appears to be an important part of silent reading (see Perrone-Bertolotti et al., 2014, for a recent review). Many people appear to evoke auditory imagery for speech while they read, and there is evidence that it retains some of the properties of external, heard speech. For instance, Alexander and Nygaard (2008) played a conversation involving two voices with different speaking rates (one fast, one slow), and then asked participants to read passages apparently written by the people whose voices they had heard. For easy texts read out loud, passages “written” by the slow voice tended to be read more slowly than those associated with the fast voice; reading silently showed no effect of voice. But for more difficult texts, both out-loud and silent reading showed evidence of being read according to the speed of speech that was previously heard. This effect also showed evidence of individual differences: those who self-reported low imagery skills only showed a voice effect on their silent reading for difficult texts, but those with high imagery skills showed the effect for easy and difficult passages of text. Thus, more complex or challenging conditions appear to prompt inner-speech-like experiences as a complementary tool during reading, but for some people this experience will persist even during easy reading.

Elsewhere, self-talk (both overt and covert) has been proposed to play a significant role in behavioral control and motivation during competition and high-performance sport (see Hardy, 2006, for a review). For instance, Hatzigeorgiades, Zourbanos, Mpoumpaki, and Theodorakis (2009) compared the effect of self-talk training on tennis players’ performance, confidence, and anxiety. Participants were randomly assigned to either three training sessions that emphasized use of motivational and instructional self-talk (e.g., “go, I can do it,” or “shoulder, low”) or control sessions that included a tactical lecture on use of particular shots. Players trained to use self-talk showed improvements in task performance (a forehand drive test), and also reported increased self-confidence and decreased anxiety, whereas no such changes were observed for the control group.

Effects of self-talk and “verbal self-guidance” are also extensive in organizational and educational psychology studies (e.g., Brown & Latham, 2006; Oliver, Markland, & Hardy, 2010), and the use of self-talk to instruct and motivate in sport and other performance-related fields is largely consistent with the view that inner speech has a primary role in self-awareness and self-evaluation (Morin, 2005, 2009a). However, research in this area has not typically distinguished between overt and covert forms of speech, making it hard to draw strong conclusions about the role that specifically internal representation of speech might play.

Nevertheless, a few recent studies have asked participants to specifically engage in imagined self-talk, and then examined the impact on motivation and behavioral control. For instance, Senay, Albarracín, and Noguchi (2010) compared the impact of interrogative and declarative self-talk on participants’ anagram performance and intention to exercise. Imagining questions in inner speech prior to starting the task (e.g., statements such as “Will I . . .?”) were associated with better anagram performance and intention to exercise compared with imagining declarative statements (e.g., “I will . . .”), with the latter being mediated by changes in the intrinsic motivation to exercise. Similar effects were found in a second study by Dolcos and Albarracín (2014) that compared inner speech in the first and second person, with prompts to imagine giving advice in the form of “You . . .” leading to better performance and motivation than imagined speech in the first person: “I can do this.” Such protocols have their limitations: they do not include a control for checking that participants were actually engaging in the kinds of self-talk they were instructed to use, nor whether participants also deployed self-talk during the subsequent performance tasks (i.e., anagrams). But they are notable for highlighting how even small changes in grammar and reference of self-talk could impact upon task motivation, and for their consistency with dialogic approaches to everyday inner speech. Indeed, Dolcos and Albarracín (2014) explicitly note that the use of second-person inner speech could reflect the putative social origins of regulatory inner speech, suggesting that “initial external encouragements expressed using *You* may become internalized and later may develop into self-encouragements” (p. 641).

Finally, the adoption of inner speech or other verbal strategies can, in some instances, be counterproductive to particular cognitive processes. The capacity for verbal labels or narratives to reshape memories and other cognitive representations has long been noted: for example, Loftus and Palmer (1974) demonstrated that the use of words like *smashed* instead of *hit* led to greater estimates of car collision speed for eyewitnesses of an accident. Verbal redescription of prior events has been most extensively studied via the phenomenon of “verbal overshadowing,” a term coined by Schooler and Engstler-Schooler (1990) following evidence that verbal description of the perpetrator of a crime was associated with a 25% reduction in recognition of the perpetrator’s face. Subsequent studies using a range of tasks have reported evidence of verbal labels appearing to reduce or distort accurate recall (Meissner & Brigham, 2001). Candidate explanations for verbal overshadowing have included interference effects from verbal content, a shift in processing focus in the translation to verbal information (from global and holistic to local and specific), and changes in decision criteria that result from verbal recoding (Chin & Schooler, 2008).

However, overshadowing effects have also proved hard to replicate, with recent studies reporting much lower effect sizes than those in Schooler and Engstler-Schooler’s original study. A recent “registered replication” attempt (Alogna et al., 2014), conducted across 31 labs, found that verbal overshadowing reduced recall by 4%–16%, depending on how close to the original event a verbal description was made. Although it is unclear how frequently and with what strength such effects occur, their existence highlights the fact that the adoption of a verbal strategy will not always be a complementary tool, and may even obscure the original representation (phonemic similarity effects following verbal recoding of visual material could also be considered an example of a maladaptive verbal strategy).

### How do Adults Experience Inner Speech?

The phenomenology of inner speech in adulthood has been investigated using two main methods: questionnaires and experience sampling. Questionnaires have the advantage of allowing data gathering from large samples in a single testing session; experience sampling, in contrast, is typically conducted with smaller numbers but can provide rich and idiographically detailed information (Alderson-Day & Fernyhough, 2014).

A variety of self-report questionnaires and listing methods have been used to assess adults’ inner speech, including the Scale for Inner Speech (Siegrist, 1995), the Self-Verbalization Questionnaire (Duncan & Cheyne, 1999), the Self-Talk Use Questionnaire (Hardy, Hall, & Hardy, 2005), and the Self-Talk Scale (Brinthaupt, Hein, & Kramer, 2009). The focus of these instruments has, however, been on the context and functions of self-talk, rather than its phenomenological properties, and they have not clearly discriminated between overt self-talk and inner speech (see Hurlburt & Heavey, 2015, for a critique).

Nevertheless, such scales shed some light on intuitive or everyday views on the functions of self-directed speech. For example, Morin, Uttl, and Hamper (2011) surveyed 380 undergraduates’ views on inner speech in an open-format procedure where participants were asked to list “as many verbalisations as they typically address to themselves” (p. 1715). Common contents of inner speech were self-addressed evaluations and emotional states, while the most common functions listed were mnemonic functions (reminders to do things) and planning. This was interpreted by the authors as supporting a primarily self-reflective role of inner speech in everyday cognition, along with its importance as a tool for thinking about the future (Morin et al., 2011). Their findings echoed earlier studies of self-verbalization, which also highlighted frequent reports of evaluative and mnemonic experiences in inner speech (Duncan & Cheyne, 1999).

Some studies have sought to explore how frequently positive and negative content occurs in self-talk, and what effect this has on other factors, such as mood. For instance, Calvete et al. (2005) developed scales of Negative and Positive Self-Talk and explored their correlates for psychopathology traits in a large sample of Spanish students (*n* = 982). The negative scale included self-talk statements about anxious, depressive, and angry self-talk, while the positive scale included items on coping, minimization of worries, and positive orientation. As might be expected, many of the positive and negative subscales were significantly associated with trait measures of psychopathology: for instance, trait depression was strongly predicted by depressive self-talk and trait anxiety by anxious and depressive self-talk. Positive predictors were more varied: minimizing inner speech was negatively associated with anxiety and anger but not depression, while positively oriented self-talk was linked to lower depression but higher levels of anger. Such results reflect the intuitive idea that inner speech is involved in the representation of everyday worries and low mood, but they also highlight a problem of construct validity: if depressive self-talk strongly predicts depressive traits, how clear is it that two separate phenomena are being measured? That is, to what extent do relations between valenced self-talk and mood reflect content overlaps in self-report measures?

The only self-report scale directly focused on the experience of inner speech is the Varieties of Inner Speech Questionnaire (McCarthy-Jones & Fernyhough, 2011). Development of the VISQ was motivated by a recognition that existing operationalisations of inner speech had been based on relatively impoverished conceptions of the phenomenon, along with an ambition to investigate aspects of inner speech, such as dialogicality and condensation, important in Vygotsky’s theory. Using data from separate exploratory and confirmatory samples of university students, factor analysis of the scale highlighted four underlying factors: *dialogic inner speech*, or the tendency to engage in inner speech with a back-and-forth, conversational quality; *condensed inner speech*, the experience of inner speech in an abbreviated or fragmentary form; *other people in inner speech* (i.e., representation of others’ voices, or inner speech saying something that someone else would usually say); and *evaluative/motivational inner speech*, where inner speech serves to judge or assess one’s own behavior. Of these, evaluative/motivational inner speech was the most commonly endorsed: 82.5% of responses indicated at least some experience of those characteristics. Dialogic inner speech was almost as prevalent (77.2%), while condensed inner speech (36.1%) and the presence of other people in inner speech (25.8%) were less common, while still being reported by a substantial minority.

Although they did not specifically ask about emotional content of inner speech, the VISQ factors also picked out tendencies toward negative emotional states: evaluative inner speech and the presence of other people in inner speech were both positively associated with trait anxiety and, to a lesser extent, depression. In a separate study (Alderson-Day et al., 2014), frequencies for the VISQ factors were closely replicated in a third student sample, and showed a further link to emotional functioning: evaluative inner speech, but not other kinds of inner speech, negatively predicted levels of global self-esteem. In addition to being specific to inner speech (rather than an unspecified mixture of overt and covert self-talk), studies with the VISQ contrast with Calvete et al. (2005) by not referring to positive or negative inner speech content directly, and yet still demonstrating links between inner speech and mood, thus avoiding concerns about content overlap.

In contrast to questionnaires, which largely focus on trait-like qualities of inner speech, experience sampling methods seek to capture moments of spontaneous experience. In one of the first studies to apply such methods to inner speech (Klinger & Cox, 1987), college students were asked to complete a short questionnaire on their inner experience following a series of random beeper alerts. Thoughts containing “interior monologue” were reported in roughly three quarters of samples, alongside regular experience of visual imagery. Experience sampling studies of inner speech since then have largely been restricted to Hurlburt’s Descriptive Experience Sampling method, which is predicated on the bracketing of presuppositions about the frequency and form of inner experience (Hurlburt & Heavey, 2006). In DES, participants only report on moments of experience that occurred immediately prior to random beep alerts (normally 1–2 s), and are encouraged to avoid generalizations about how they usually think or “what they always do.” Hurlburt, Heavey, and Kelsey (2013) argue that one result is that DES provides a more accurate indication of the frequency of inner speech, and that generally this is much lower than other estimates, occurring in around 20%–25% of random samples (although see Alderson-Day and Fernyhough, 2014).

In addition, DES has provided an exceptionally rich body of data on the many forms that inner speech can take (Hurlburt et al., 2013). Preferring the term *inner speaking* to *inner speech* (in order to emphasize its active nature), Hurlburt et al. note several key features of the phenomenon: individuals typically apprehend themselves to be speaking meaningfully in the absence of vocalizations; these experiences are generally in the person’s own voice, with its characteristic rhythm, pacing, tone, and so forth; the utterances are similar in form to external speaking, and bear the same potential emotional weight; inner speaking is generally in complete sentences, uses the same kinds of words as external speech, and can be addressed either to the self or to another; and the phenomenon is apprehended as being actively produced rather than passively heard.

A distinct form of inner experience, not reducible to inner speaking, is *inner hearing*, which Hurlburt defines as “the experience of hearing something that does not exist” in the individual’s immediate surroundings or external environment (p. 1485). Other categories of inner experience that are not equated to inner speaking are *unsymbolized thinking*, or “the experience of an explicit, differentiated thinking that does not include words, images, or any other symbols” (p. 1486), *sensory awareness*, and *thinking* (defined as a purely cognitive process without any phenomenological qualities).

Finally, in a study that could be seen as occupying a middle ground between questionnaire-based studies and experience sampling, D’Argembeau, Renaud, and Van der Linden (2011) conducted a thought diary experiment, where participants were asked to keep track of any future-directed thoughts they had over the course of a day. Recorded thoughts (written in a notebook) were rated by participants for a variety of characteristics, such as modality (e.g., inner speech, visual), affective content, and personal importance, and coded by experimenters for function, time specificity, and valence. Experiences of inner speech were particularly associated with action-planning and decision-making, in contrast to more visual forms of future-oriented cognition. In such cases, the everyday phenomenology of inner speech appears to parallel its accompaniment to specific cognitive tasks where inner speech is used as a planning or deliberative tool.

### What is the Relation Between Inner Speech and Overt Speech?

Examining the relation between inner speech and its overt counterpart can enable the testing of models of inner speech production. Recall that one model, often associated with Watson (1913), holds that inner speech is identical to external speech with highly attenuated articulatory commands. A contemporary version of this model, the *motor simulation* hypothesis, is an example of a wider group of “embodied simulation” theories (e.g., Bergen, 2012), which hold that processes such as word understanding and mental imagery have similar content and structure to actions or perceptions but attenuated characteristics. On such a model, inner speech and overt speech should share a number of linguistic and structural features. In contrast, views of inner speech (such as Vygotsky’s) that see it as representing a transformed version of external speech would predict that inner speech would lack the featural richness of overt speech, and may vary in form depending on context (thus avoiding the processing costs of vividly representing speech on each occasion). For instance, Fernyhough (2004) proposed that inner speech varies with cognitive and emotional conditions between abstracted (*condensed*) and concrete (*expanded*) forms.

Researchers have addressed the question of the phenomenological richness of inner speech by studying errors and delays in its production. In a silent reading study, Filik and Barber (2011) compared eye movements in participants with northern or southern English accents when reading limericks. The poems were designed to either rhyme or clash in the participants’ normal accent (e.g., *mass* and *glass* rhyme in a northern English accent, but not a southern accent). Compared with congruent poems, limericks that did not rhyme in the participant’s accent led to disruption in eye-tracking patterns, suggesting that participants’ inner speech retained the surface-level auditory properties of their external speaking voice.

A contrasting view is provided by Oppenheim and Dell (2008), who have argued that inner speech differs from overt speech in many of its psycholinguistic properties. Specifically, they argue that inner speech retains deep features, such as lexical and semantic information, but typically does not represent surface-level information such as phonological detail. Their evidence comes from a comparison of tongue-twister errors in overt and inner speech, in which participants report on the internal errors that they make. While in overt speech errors occurred reflecting both lexical bias (the tendency to produce a real word rather than a nonword), and phonemic similarity effects (such as substituting *reef* and *leaf*), in inner speech only the former were reported. Oppenheim and Dell interpreted this as evidence that inner speech is impoverished at featural levels.

In contrast, two studies by Corley and colleagues reported similar phoneme substitution errors in inner and overt speech, for both fluent speakers (Corley, Brocklehurst, & Moat, 2011) and adults who stutter (Brocklehurst & Corley, 2011). Making phoneme substitutions in inner speech would suggest that specific phonological features are encoded in inner speech and available to internal inspection. Such findings support a common view in psycholinguistic research that inner speech largely serves to support error monitoring in speech production, whereby utterances can be inspected and corrected via an “internal loop” (e.g., Wheeldon & Levelt, 1995).

One way to reconcile these varied findings on the phenomenological richness of inner speech is to consider how it might be affected by articulation. In follow-up work, Oppenheim and Dell (2010) showed that phonemic similarity errors do appear if participants perform the tongue-twister task with the addition of silent mouthing, but not if participants are instructed to imagine saying phrases “without moving their mouth, lips, throat, or tongue” (Oppenheim & Dell, 2010, p. 1552). These findings led Oppenheim and Dell to propose the *flexible abstraction hypothesis*, according to which there is only one kind of inner speech, represented at the level of phonemic selection, but where that representation can be modulated by articulation to include more explicit features. Thus, in cases where inner speech appears to have specific phonological features (as in Corley et al., 2011), this may have been be due to participants deploying a form of inner speech involving a greater degree of articulation (such as silently mouthing words as they are represented in inner speech).

The reliance on participant self-report for errors in inner speech is an important limitation when interpreting these studies. As Hubbard (2010) has argued, apparent differences in phonological features between overt and covert speech may simply reflect participants’ ability to introspectively monitor and report specific features of their inner speech. However, this would not explain the presence of similar features in overt and covert speech in Corley, Brocklehurst, and Moat’s (2011) study, or when a greater level of articulation is deployed (Oppenheim & Dell, 2010).

Moving beyond inner speech production to the processes involved in generating external speech, there is a large body of psycholinguistic research on the role of inner speech as a potential error monitor for external speech (e.g., Hartsuiker & Kolk, 2001; Nooteboom, 2005), a full discussion of which is beyond the scope of this article (see Hickok, 2012, for a review). Key to most of such models is that inner speech is posited as part of a speech production system involving predictive simulations or “forward models” of linguistic representations. Such forward models prepare perceptual systems for self-generated inputs: for example, producing overt speech is thought to involve the sending of an “efference copy” of the speech motor plan to speech perception areas, forming the basis for a predictive model of what the utterance will sound like, and inhibiting the ensuing auditory response (Grush, 2004).

Where inner speech fits in to such models is not always clear, not least because there appears to be no external percept or motor consequence to be attenuated if no sound is created. Producing inner speech can have similar influences to overt speech on speech perception, such as priming perception of external sounds (e.g., Scott, Yeung, Gick, & Werker, 2013), suggesting that it too involves the sending of efference copies to receptive areas. One possibility is that inner speech is a minimal form of overt speech that has been attenuated because it is recognised as being self-produced (for a discussion of this possibility, see Langland-Hassan, 2008). Alternatively, it has been suggested that inner speech in some way constitutes a featurally abstract forward model (Pickering & Garrod, 2013), or that we experience phonological features in inner speech *because of* the sensory prediction created by a forward model (Scott, 2013). As will be discussed in the final section, this has implications for models of auditory verbal hallucinations in which inner speech is proposed to be misattributed to an external source.

### Inner Speech in the Brain

The similarities and differences between inner speech and external speech have also been examined in relation to underlying neural processes. Research in this area has come from studies on speech-motor processing in the brain, which has largely treated inner speech as a covert articulatory planning process (for a review, see Price, 2012), researchers interested in inner speech dysfunction as a basis for psychopathology (McGuire, Murray, & Shah, 1993; Shergill, Brammer, Williams, Murray, & McGuire, 2000, see Adult Psychopathology), and work on the rehearsal and maintenance of verbal working memory (e.g., Marvel & Desmond, 2010).

A *prima facie* assumption might be that the neural correlates of inner speech would simply reflect an attenuated or inhibited version of neural states associated with overt speech. In support of this, activation of Broca’s area or left inferior frontal gyrus has been observed during both overt and silent articulation of words, specifically in the ventral portion of the pars opercularis (Price, 2012). Alongside this, supplementary motor area (SMA) and parts of premotor cortex are often implicated, in addition to the anterior portion of the insula, although it has been claimed that the latter is more specifically tied to muscular processes required for overt speech production (Ackermann & Riecker, 2004).

Based on evidence from neuropsychological studies, it has been argued that verbal working memory processes rely on a separate neural network to speech production (Baddeley & Logie, 1999; see The Neuropsychology of Inner Speech). However, most recent studies have implicated similar and overlapping networks for verbal working memory maintenance and overt speech in fronto-temporal regions, along with recruitment of the cerebellum (Marvel & Desmond, 2012) and posterior temporoparietal structures such as the planum temporale and inferior parietal lobule (Andreatta, Stemple, Joshi, & Jiang, 2010). While the cerebellum is thought to support motor processes involved in verbal rehearsal (Marvel & Desmond, 2010), the involvement of temporoparietal cortex has been proposed to reflect recruitment from long-term memory of phonological representations to support working memory maintenance (Price, 2012). Activation of inferior frontal gyrus (IFG), premotor cortex, and the Sylvian parietal-temporal area (SPT) show both load and rehearsal rate effects during verbal working memory maintenance (Fegen, Buchsbaum, & D’Esposito, 2015), while disruption to posterior superior temporal gyrus using repetitive transcranial magnetic stimulation interferes with both speech production and verbal working memory maintenance (Acheson, Hamidi, Binder, & Postle, 2011).

The concurrent recruitment of inferior frontal and posterior temporal regions during inner speech is supported by earlier studies of covert speech, auditory imagery, and verbal self-monitoring. McGuire et al. (1996) asked participants either to articulate sentences silently from cue words, or to imagine them in another’s voice. (In order to distinguish it from inner speech, the latter was referred to as *auditory verbal imagery*.) Contrasts using PET scanning indicated that inner speech was associated with left IFG activation, while imagining another’s speech involved SMA, premotor cortex, and left superior and middle temporal gyri. As these temporal areas in particular are typically associated with speech perception, the authors suggested that this reflects a greater “internal inspection” during the generation of representations of others’ speech, driven by the need to pay particular attention to representing the phonological characteristics of another’s voice. Subsequent research has also implicated similar regions of temporal cortex in the monitoring of inner speech: Shergill et al. (2002), for instance, reported greater activation of superior temporal gyrus, left IFG, and the pre- and postcentral gyri when participants were asked to vary the speed of their inner speech.

One problem with such studies is the lack of a behavioral control when asking participants to generate inner speech in the scanner. As noted in the auditory imagery literature (Hubbard, 2010; Zatorre & Halpern, 2005), it is risky to rely on participants’ own reports of inner speech, even if the areas identified in such studies appear to coincide with speech production networks. One way of avoiding this is to use inner speech tasks that rely on phonological judgments. For instance, Aleman et al. (2005) used fMRI to scan participants while they either listened to or imagined hearing words that were pronounced with the stress on the first or second syllable. For both heard and imagined speech, inferior frontal gyrus, insula, and superior temporal gyrus were activated, although for the latter region only a posterior portion was active for imagined words. As this pattern of activity was not seen for a comparable task where participants had to make a semantic judgment about the words, Aleman and colleagues argued that posterior superior temporal gyri (STG) was required for representation of metric stress in the phonological loop. This, when combined with evidence from studies of verbal working memory, would seem to support the general fronto-temporal network of areas highlighted in inner speech elicitation studies (e.g., McGuire et al., 1996), notwithstanding their lack of behavioral controls.

Another concern about standard neuroimaging approaches to inner speech is that they are limited by the temporal resolution of fMRI, meaning that the dynamic interplay between areas responsible for speech production and perception may be overlooked. Neurophysiological techniques, such electroencephalography (EEG) and magnetoencephalography (MEG), offer millisecond-scale resolution, albeit usually at the expense of spatial precision within the brain.

Preliminary evidence from MEG research has highlighted potential differences in the timecourse of different kinds of inner speech, and how its production affects speech perception areas in temporal cortex. Tian and Poeppel (2010) compared MEG responses for (a) overt articulation, (b) imagining saying something in one’s own voice, (c) imagined hearing something in someone else’s voice, and (d) actually hearing another’s voice. Imagined speaking and hearing both localized to bilateral temporal cortex (which was interpreted as indicating the auditory simulation process), but imagery for speaking localized first to left parietal cortex (Tian & Poeppel, 2010). In a subsequent experiment, imagery for speaking and hearing appeared to have different repetition priming effects on auditory cortical responses: the former increasing activity, and the latter inhibiting it (Tian & Poeppel, 2013). Tian and Poeppel argue that these differences exist because the additional motor elements of imagined speech involve the deployment of a somatosensory forward model (i.e., not just a sensory simulation), and serve to prime auditory areas to recognise a given response (a top-down effect), rather than habituate them to an old response (a bottom-up effect; Tian & Poeppel, 2012). The involvement of parietal cortex is also consistent with findings from studies of mental imagery in other modalities, which often involve recruitment of the superior and inferior parietal lobules (McNorgan, 2012).

One caveat in interpreting Tian and Poeppel’s findings is that they compare imagined speaking in one’s own voice with imagined hearing of another’s voice, making it hard to disentangle additional demands involved in generating one’s own voice versus another’s (cf. McGuire et al., 1996). In addition, they explicitly refer to their stimuli as prompting mental imagery, rather than inner speech, leaving open to what extent their task is tapping similar processes to those involved in verbal rehearsal, for example. Nevertheless, the suggested separation of “spoken” and “heard” representations in their results would be consistent with separable articulatory rehearsal and phonological store components in the phonological loop (Baddeley & Logie, 1999). They are also in line with separate behavioral effects of the “inner voice” and “inner ear” that have been reported in auditory imagery experiments (Hubbard, 2010), and Hurlburt’s distinction between inner speaking and inner hearing (Hurlburt et al., 2013).

While the above findings are informative about the neural components of inner speech, one final concern about such studies is their ecological validity. Many have largely relied on relatively simple word- or sentence-repetition paradigms, meaning that they may miss a degree of complexity and variety inherent in everyday inner speech (Jones & Fernyhough, 2007). Some recent studies have reported on the neural basis of more naturalistic forms of inner speech, such as those involved in silent reading, or spontaneous cognition during verbal mind-wandering (also known as *stimulus-independent thought*). For instance, Yao, Belin, and Scheepers (2011) compared brain activation during reading for direct speech (*The man said “Get in the car”*) and indirect speech (*The man said to get in the car*), on the rationale that the former likely involved specific representation of a character’s voice. Compared with passages of indirect speech, direct speech was associated with greater activation in right auditory cortex (posterior and middle superior temporal sulcus), alongside recruitment of the superior parietal lobules, precuneus, and occipital regions bilaterally. The authors argued that this reflects a more vivid perceptual simulation of the “inner voice” during reading, in a way that might be more spontaneous and ecologically valid than methods that require the top-down elicitation of specific voices in inner speech (cf. Shergill et al., 2001).

A second example is provided by Doucet et al. (2012), who studied self-reported inner speech during a resting-state MRI session. A large sample of participants (*n* = 307) completed a custom-designed questionnaire (Delamillieure et al., 2010) about their resting cognition immediately after an 8-min scan. Participants’ reports for proportion of time spent in either inner speech or visual imagery were then assessed for their effect on connectivity within five resting brain networks selected using independent components analysis (ICA). Greater time spent using either inner speech or visual imagery was linked to reduced connectivity between two networks: the default mode network, which is usually associated with introspection and self-referential thinking (Raichle et al., 2001), and a fronto-parieto-temporal network, including the inferior frontal gyrus, middle and inferior temporal gyri, angular gyrus, and precuneus. Fronto-parietal networks are often thought to support attentional focus and engagement, and in a prior study Doucet and colleagues had linked this fronto-parieto-temporal network to the maintenance of internally generated representations (Doucet et al., 2011). Thus, the use of either inner speech or visual imagery in this case appeared to involve some sort of decoupling between introspective and attentional processes. Although these data are only preliminary and not specific to inner speech, they point toward the possibility of identifying separable resting networks involved in the generation and maintenance of spontaneous verbal thoughts.

### Inner Speech and Variations in Linguistic Experience

One further difference between the developmental and working memory approaches to inner speech is in their relative emphasis on the influence of linguistic experience. Specifically, the Vygotskian developmental account would hold that variations in language experience should be reflected in the subsequent nature of self-directed speech. In private speech research, this idea has been tested by examining the effect of, for example, culture-specific patterns of child–adult interaction (Berk & Garvin, 1984) and contrasts between collectivistic and individualistic cultures (Al-Namlah et al., 2006). Perhaps reflecting the greater methodological challenges in studying speech that has been fully internalized, there are very few studies that speak to this topic in relation to inner speech.

Research on bilingualism has provided some preliminary information on the relation between inner speech and prior linguistic experience, largely via studies of second language (L2) learning. In general, use of L2 inner speech appears to increase with proficiency in the second language, but also evidences a change in function, with less fluent learners reporting use of L2 specifically for rehearsal and planning of speech, but more able speakers using it for less voluntary and more abstract modes of thinking (de Guerrero, 2005). L2 learners have also been known to report a growing “din” of sounds and words from the second language in their mental experience as they become more proficient (Krashen, 1983), an experience that is suggestive of the internalization or developing automaticity of thought in L2.

There is also evidence that L2 learning may have a differing impact upon inner speech and related processes depending on when it is encountered. For instance, Larsen, Schrauf, Fromholt, and Rubin (2002) studied inner speech and autobiographical memory in relation to second-language learning among Polish immigrants living in Denmark. Half of the participants were “early” immigrants, moving at the average age of 24, while the other half had moved at a later age (*M* = 34 years). Despite both groups having lived in Denmark for at least 30 years, early compared with late immigrants reported greater use of Danish inner speech, while both groups tended to report autobiographical memories in Polish when the recalled events occurred prior to moving, and in Danish when the events occurred after moving. Two implications can be drawn from this study: first, that the language of inner speech is affected by the age of acquisition of a second language, and second that any such effect may be independent of a language-specificity effect linking recall of autobiographical memories to the language used at encoding.

Another approach to this question is to consider the experience of inner speech, or analogous processes such as imagery for sign language, in people who are deaf. Historically, a large body of psychological research was conducted under the mistaken assumption that people who are deaf would have no inner language facility, and would thus lack certain capacities for abstract thought (see, e.g., Oléron, 1953). This not only assumed an identity between language and complex thought, but also failed to recognise deaf people’s ability to use nonspeech based languages, such as signing. This impoverished view of deaf individuals’ cognition only began to be overturned in the 1960s, with the emergence of studies reporting abstract, nonverbal reasoning in deaf individuals (e.g., Furth, 1964) and a rise in awareness that sign-based languages are highly rich and complex languages in their own right (Stokoe, 2005).

Since then, a broad range of studies have examined verbal and nonverbal cognitive skills in deafness (see Marschark, 2006, for a review), although still very little is known about the use and prevalence of inner speech or sign by deaf people. From a developmental perspective, it may be expected that deaf individuals would report qualitatively different experiences of their inner speech, or be less likely to engage in certain kinds of inner speech, if their opportunities to engage communicatively with others in early childhood are constrained (over 90% of deaf children have hearing parents; Vaccari & Marschark, 1997). However, recent data from a questionnaire study on private sign and inner speech by Zimmermann and Brugger (2013) suggest the opposite. In a sample of 28 hearing and 28 deaf adults (of whom 20 were congenitally deaf), Zimmerman and Brugger reported regular use of “signed soliloquy”—overt signing for a private purpose—in deaf signers, which occurred with a greater frequency than did private speech in hearing participants. In addition, deaf participants reported greater use of positive/motivational “inner speech” compared with hearing participants, although the questionnaire used to measure this, the Inventory on Self-Communication for Adults, did not ask participants to distinguish whether this was a specifically verbal or signed experience. The authors interpreted both of these findings as reflecting possible use of coping strategies to counteract feelings of isolation associated with the experience of hearing impairment.

These findings point to the importance of conducting more research with larger samples of deaf individuals, and particularly the necessity of examining the influence of differing linguistic backgrounds, which in the deaf population can be very heterogeneous. That said, existing findings are at least suggestive of the possibility that inner speech or other forms of self-directed language can form part of positive compensatory strategies rather than merely being shaped by prior social interactions.

A final group of interest in this regard is adults who for various reasons have poor language skills. Alarcón-Rubio, Sánchez-Medina, and Winsler (2013) studied private speech use in illiterate adults, in comparison with those with high literacy, when engaging with a categorization task. Compared with high-literacy participants, participants with low literacy displayed much more externalized private speech, particularly on more difficult forms of the task. Such findings support the Vygotskian prediction that linguistic skills are associated with a general internalization of verbal strategies.

## Inner Speech in Atypical Populations

The foregoing review has demonstrated that inner speech plays a prominent role in everyday experience and cognitive function for healthy children and adults. Important information on the psychological significance of inner speech is also provided by studies of how typical processes of inner speech development and production are perturbed in atypical populations, including developmental disorders and psychiatric illnesses in adulthood.

### Developmental Disorders

One area that has seen an increased attention to inner speech is the study of autism spectrum disorders (ASDs). ASDs are characterized by difficulties in social interaction and communication alongside the presence of restricted interests and repetitive behaviors (WHO, 1993). Many children with an ASD show delays in early language development, and even those with good structural language skills—such as children with Asperger syndrome (AS)—typically have enduring difficulties in communicating with others. Given the proposed grounding of inner speech in external communication and interaction, it follows that the development of inner speech is likely to be disrupted and/or delayed in individuals with an ASD (Fernyhough, 1996). This in turn could have implications for the understanding of cognitive strengths and weaknesses seen in ASD, such as problems with theory of mind and executive functioning skills (Baron-Cohen, Leslie, & Frith, 1985; Russell, 1997).

Anecdotal support for this idea comes from the descriptions of inner experience made by people with ASDs. Most notably, Temple Grandin (1995) is known for describing her experience of thought as “thinking in pictures” rather than inner speech (for an elaboration of this idea, see Kunda & Goel, 2010). In a study using DES, Hurlburt, Happé, and Frith (1994) interviewed three adults with AS about their inner experience. As the authors noted, there are questions about the communicative and introspective demands of this technique for individuals with ASD. Nevertheless, two of the three participants reported uniformly visual experiences, and none of the three described experiences of inner speech or internal dialogue (Hurlburt et al., 1994).

A number of subsequent experimental studies have examined inner speech in autism, primarily using paradigms from executive functioning research. Inner speech was indirectly probed by Russell, Jarrold, and Hood (1999) in a study of executive skills in children with autism and typically developing matched controls. Two tasks were deployed which either (a) did not require the maintenance of an explicit rule, or (b) demanded an overt verbal response that would conflict with maintenance of inner speech. ASD participants showed intact performance on both tasks, in contrast to evidence of executive difficulties in other studies on autism (Hughes, Russell, & Robbins, 1994; Ozonoff, Pennington, & Rogers, 1991), which the authors argued could reflect differences in the deployment of inner speech in ASD. On the first task, no specific rule needed to be maintained in inner speech, leading to equal performance in both groups. On the second task, the requirement to respond verbally could have produced a conflict for typically developing participants if they were also maintaining a rule in inner speech, thus nullifying any advantage they might have had over participants with autism (Russell et al., 1999).

Whitehouse, Maybery, and Durkin (2006) conducted the first study directly examining inner speech in ASD. On a verbal recall task, children with ASD showed a reduced picture superiority effect compared with controls—an effect which is thought to rely on dual coding of pictures visually and verbally via inner speech (Paivio, 1991). In follow-up experiments, the same group of participants showed a diminished word-length effect on their verbal recall, and no effect of articulatory suppression, both of which suggested a diminished use of inner speech to support memory processes (Whitehouse et al., 2006).

Further evidence of irregularities in inner speech was provided in studies by Holland and Low (2010), Russell-Smith, Comerford, Maybery, and Whitehouse (2014), and Wallace et al. (2009). Wallace et al. (2009) compared problem-solving performance on the Tower of London with and without articulatory suppression in adolescents with autism and typically developing controls. Pairwise comparisons of performance in each group indicated that typically developing participants, but not ASD participants, were adversely affected by articulatory suppression, suggesting an interference effect with inner speech. It should be noted, however, that the initial group-by-condition interaction effect was not significant in this case, and the main effect of group only approached significance (Wallace et al., 2009). Holland and Low (2010) also compared children with autism and typically developing controls on a towers task (the Tower of Hanoi) along with an arithmetic-based switching task. On both tasks, children with autism were affected less by articulatory suppression than were control children, and on the arithmetic task children with autism also showed proportionately greater interference from a visuospatial distractor activity. Finally, Russell-Smith et al. (2014) compared children with ASD and typically developing children on a card-sorting task under normal conditions, articulatory suppression, explicit strategy verbalization, and concurrent mouthing (included to control for nonspecific motor demands). Articulatory suppression impaired performance in typically developing children, but not ASD children. Moreover, explicit verbalization—which may have been expected to benefit the ASD group if they were not already using inner speech—only showed benefits for control participants. Thus, across tasks drawing on capacities for memory, planning, and cognitive flexibility, there is evidence that inner speech is less likely to be used by children with ASD than by their typically developing counterparts.

However, evidence of typical verbal strategy use in ASD children has also been reported in some cases (Williams, Happé, & Jarrold, 2008; Winsler, Abar, Feder, Schunn, & Rubio, 2007). In a study contrasting children with autism, children with attention deficit hyperactivity disorder (ADHD), and typically developing children, Winsler, Abar, Feder, Schunn, and Rubio (2007) coded overt private speech use on the Wisconsin Card Sorting Task (Heaton, 1993) and a physical problem-solving task. Contrary to expectations, no consistent group differences were observed in private speech use, with around 70% of ASD participants spontaneously using private speech to support their performance. As no interference tasks were used, the findings do not show that internalized verbal strategies (i.e., inner speech) were being used in the same way, but they are suggestive of similarities in inner speech use between ASD, ADHD, and typically developing children.

Supporting this idea, Williams, Happé, and Jarrold (2008) reported intact use of inner speech during verbal recall in children with autism. Using a task that included pictures that were either phonologically similar, visuospatially similar, or dissimilar in both respects, both ASD and control children showed evidence of the phonological similarity effect, proposed to occur when inner speech is used to recode pictures into words to assist recall.

Williams and colleagues argued that these results reflect intact inner speech as a mechanism to support recall in ASD, but did not rule out potential qualitative differences in inner speech. One way in which inner speech in autism could differ qualitatively from inner speech in typical development is in the resources drawn on to support it. Lidstone, Fernyhough, Meins, and Whitehouse (2009) conducted a reanalysis of the data from Whitehouse et al. (2006) comparing relations between cognitive profile and inner speech in children with autism and in typically developing controls. Because inner speech is proposed to have a basis in early communicative interaction, Lidstone and colleagues hypothesized that children with autism with greater nonverbal than verbal skills (a cognitive profile common in ASD) would also be less likely to use inner speech during task performance. This prediction was confirmed: only ASD participants showed a significant effect of cognitive profile, with NV > V participants showing the least interference from articulatory suppression on an arithmetic switching task. The authors also suggested that this may explain some of the previous null findings of inner speech differences in autism reported by Williams et al. (2008). This, however, was not supported in a reanalysis of the Williams et al. (2008) data by Williams and Jarrold (2010), who found verbal ability to be the strongest predictor of inner speech use, rather than the relative levels of verbal and nonverbal skills.

Qualitative differences in inner speech in ASD might also be evidenced in the formal properties of inner speech. Williams, Bowler, and Jarrold (2012) studied inner speech use in adults with ASD on a verbal recall task and a Tower of London planning task. On the former, the phonological similarity effect and articulatory suppression effect were used as indices of inner speech use; on the latter, the index was the size of the articulatory suppression effect. On the memory task, both ASD and typically developing adults showed evidence of inner speech use, but on the planning task only controls were affected by articulatory suppression.

Williams et al. argued that sense could be made of these results by drawing on Fernyhough’s (1996) distinction between monologic and dialogic inner speech. The memory task only requires verbal material to be rehearsed, via repetition, in a way that does not require the coordination of multiple perspectives: in other words, a monologic strategy. In contrast, the planning task required a dialogic consideration of multiple alternatives and routes, and the weighing-up of different strategies. If dialogic thinking (Fernyhough, 1996, 2009a) has its roots in external communication and interaction with others, then it is dialogic but not monologic inner speech that would be expected to be either atypical or absent in ASD. Thus, it could be that ASD individuals deploy monologic inner speech to support their cognitive performance, but either do not possess or do not use dialogic inner speech in the same way (Williams et al., 2012). Supporting this idea, communication scores for ASD participants on the Autism Diagnostic Observation Schedule (ADOS; Lord et al., 2000) and Autism Quotient (Baron-Cohen, Wheelwright, Skinner, Martin, & Clubley, 2001) were observed to predict articulatory suppression effects during planning, suggesting a link between communicative ability and lack of inner speech specifically to support problem-solving. Further work is needed to test this hypothesis: the same distinction has not yet been tested in children with ASD, nor on other problem-solving tasks that would in theory require dialogic inner speech. Nevertheless it represents a promising route for understanding inner speech in a unique population.

Similar benefits of studying atypical populations emerge in the example of specific language impairment (SLI). Lidstone et al. (2012) proposed that, if inner speech use is related to earlier communicative development, children with an SLI may be expected to demonstrate delay or deviation in their inner speech skills. In line with the evidence of private speech use in adults with literacy problems (Alarcón-Rubio, Sánchez-Medina, & Winsler, 2013), children with an SLI showed normal effects of articulatory suppression on a towers task, but evidenced less internalized forms of private speech while attempting the task. Lidstone and colleagues interpreted their results as an example of delayed inner speech internalization, rather than a qualitative difference in verbal strategy use. Evidence of general delays or disruptions to self-directed speech as a result of developmental disorder is also provided by research on ADHD (Corkum et al., 2008; Kopecky, Chang, Klorman, Thatcher, & Borgstedt, 2005), although thus far these studies have only reported on private speech, rather than inner speech.

The identification of differences in self-directed speech in developmental disorders raises the prospect of developing training and instruction methods that could benefit those with cognitive or behavioral difficulties. In the case of autism, it may be that encouragement to engage in dialogic speech processes such as asking questions or taking different perspectives could benefit individuals’ performance on specific tasks or in certain scenarios. However, it is important to recognise that the use of differing cognitive strategies in this group is a mark of variation, not deficiency: the adult participants with ASD in Williams et al.’s (2012) study could complete tower tasks apparently without recourse to verbal strategies, so intervention in this case would be inappropriate. Where training with verbal protocols may be more warranted is in situations that demand specific use of verbal strategies: for instance, use of written cues improves problem-solving efficiency on the Twenty Questions task in children with ASD (Alderson-Day, 2011). In the cases of SLI and ADHD, instructional training in private speech at earlier ages may serve to counteract apparent delays in verbal strategy use. “Think Aloud” methods have been used for some time with children with specific educational needs (e.g., Montague & Applegate, 1993; Rosenzweig, Krawec, & Montague, 2011), although such methods have been criticized in the past for being overly instructional and failing to recognise that children’s own strategies need to be facilitated, rather than being prescribed by another (Diaz & Berk, 1995). As with ASD, the exact kind of training required will likely depend on the specific skills of the child and their ability to engage in a social and scaffolded process. One promising avenue of research here is the use of microanalytic methods to study exactly when within tasks different kinds of self-talk are deployed (see Kuvalja, Verma, & Whitebread, 2014, for a recent example of such research in an SLI sample).

### Adult Psychopathology

Atypical processing of inner speech has been implicated in psychotic disorders, mood disorders, and anxiety disorders. In relation to psychotic disorders, inner speech has been particularly strongly associated with the phenomenon of auditory verbal hallucinations (AVHs), or the experience of hearing a voice in the absence of any speaker. AVHs—also sometimes referred to as “voice-hearing” experiences—are typically associated with the diagnosis of schizophrenia, but are by no means limited to that group of disorders and occur in a significant minority of the general population as well (Johns et al., 2014). A prominent theory of AVHs holds that they stem from misattribution of inner speech to an external source (Bentall, 1990; Feinberg, 1978; Frith, 1992). This model has received some support from cognitive studies demonstrating self- and source-monitoring deficits in individuals who experience AVHs (Brookwell, Bentall, & Varese, 2013; Waters, Woodward, Allen, Aleman, & Sommer, 2012).

The inner speech model of AVHs also gains support from neuroimaging studies showing activation of language networks during AVHs (Allen et al., 2012). Findings from “symptom-capture” studies (investigating neural correlates of the occurrence of AVHs in the scanner) show activation of inferior frontal gyrus bilaterally (Kühn & Gallinat, 2012), while speech-processing atypicalities in schizophrenia patients who experience AVHs are also consistent with a model in which self-generated speech is likely to be misattributed (Ford & Mathalon, 2004; Whitford et al., 2011). Finally, results from neurostimulation studies point to activation of language-relevant areas in AVHs but also highlight inconsistencies requiring refinement of the standard inner speech model of voice-hearing (Moseley, Fernyhough, & Ellison, 2013).

Despite the support for an inner speech account of AVHs, several outstanding difficulties remain in accounting for voice-hearing in terms of inner speech. One relates to the difficulty of studying the state of AVH during scanning via symptom-capture studies (Ford et al., 2012). Recent meta-analyses of symptom-capture studies have come to only partially overlapping conclusions: while Jardri et al. (2011) found AVHs to be associated with activation in left IFG, anterior insula, superior temporal, and hippocampal areas, Kühn and Gallinat (2012) could only find consistent results for bilateral IFG, postcentral gyrus, and parietal areas. The involvement of left IFG in both analyses appears to implicate Broca’s area in the AVH state, but the lack of overlap in other areas precludes inferences about how exactly inner speech may come to be experienced as having an external source. Furthermore, there is evidence that the observed Broca’s area activation could be an artifact of the target detection demands involved in many symptom-capture designs: other stimulus detection tasks involving the monitoring of a particular target and a button press to indicate its presence also often activate this brain region, and it is possible that more naturalistic or retrospective forms of symptom capture would reveal more consistent results for alternative regions (van Lutterveld, Diederen, Koops, Begemann, & Sommer, 2013; although see Shergill et al., 2000). As such, evidence from neuroimaging research is suggestive of inner speech being involved in the occurrence of AVHs, but problems in interpreting the evidence remain.

Attempts to evaluate the inner speech model of AVHs have also been limited by impoverished conceptions of inner speech and inappropriate methods for eliciting it (Jones & Fernyhough, 2007; Moseley & Wilkinson, 2014). An emerging competitor account conceptualizes AVHs as intrusions from memory, a view arguably supported by evidence of aberrant hippocampal activations in AVHs (van Lutterveld et al., 2012). A further development has been the recognition that AVHs likely take multiple forms (Jones, 2010), with only some forms of the phenomenon being explicable in terms of misattributed inner speech: others may be better understood in terms of a “hypervigilant” attention to external threat (Dodgson & Gordon, 2009), or intrusions from memory (Michie, Badcock, Waters, & Maybery, 2005). Finally, the inner speech model is arguably only applicable to AVHs, not to hallucinations in other modalities, such as visions.

Despite these concerns, the inner speech account of AVHs remains a powerful explanatory tool for at least some voice-hearing experiences, and one that is worthy of further investigation. Phenomenologically, AVHs bear many important resemblances to the experience of typical inner speech (Larøi et al., 2012), such as their frequent dialogicality and self-regulatory quality. Areas of active research interest include understanding the relation between clinical and nonclinical AVHs (Johns et al., 2014), including findings that AVHs in nonpatients are associated with more typical neural organization of language processes than in clinical groups (Diederen, De Weijer, et al., 2010). One possibility is that the distinction between clinical and nonclinical AVHs relates to differing roles of stress and cognitive challenge in triggering anomalous attributions of inner speech (Fernyhough, 2004). Subclinical hallucinatory experiences may also relate to specific characteristics of inner speech in the nonclinical population: dialogic and evaluative characteristics of inner speech, along with the presence of other people in inner speech, have all been related to auditory hallucination proneness in undergraduate samples (Alderson-Day et al., 2014; McCarthy-Jones & Fernyhough, 2011).

The role of inner speech in hallucinatory experiences is further illuminated by the example of AVHs in deafness (Atkinson, Gleeson, Cromwell, & O’Rourke, 2007; Pedersen & Nielsen, 2013). Some people who are deaf either prelingually or from birth have reportedly had the experience of “hearing” voices in the absence of a speaker (e.g., du Feu & McKenna, 1999). Close examination of the phenomenology of such experiences, however, suggests that they rarely incorporate explicit auditory properties. Rather, prior reports may reflect misinterpretation of patients’ descriptions by (predominantly hearing) practitioners and researchers, differing usages of terms such as “loudness” across spoken and signed languages, or deployment of hallucination scales and interviews that do not translate well into use with the deaf population (Atkinson, 2006). When custom-made materials that are specifically designed for deaf participants are used to enquire about unusual experiences, a wide variety of primarily communicative, but not necessarily auditory, phenomena are reported, including experiences of fingerspelling, subvocal sensations, and visual experiences of signing and lipreading. Furthermore, they appear to broadly map on to the prior linguistic experience of the individual: those who had experience of spoken language prior to hearing loss reported more auditory hallucinatory phenomena, while those with little or no access to spoken or signed languages in early childhood reported nonverbal communicative sensations that appeared to lack a specific auditory, visual, or tactile modality (Atkinson et al., 2007).

The range of experiences described in such reports, and their implications for self-monitoring accounts of inner speech and AVHs, make it tempting to draw a range of conclusions. First, it could be argued that the evidence for the existence of AVHs in deaf individuals implies that a misattribution of inner speech is less important to explaining the phenomenon than a more general misattribution of a communicative or articulatory code: that is, it would appear to force a generalization of the self-monitoring account of AVHs, beyond the specifics of speech and into a more general conception of communication. Second, if the deaf and hearing experience of AVH were considered to be comparable, and AVH in deaf participants reflected their prior linguistic experience, then the same might also be expected of AVH and inner speech in the hearing population. Inner speech on this reading would be an internalized reflection of prior communicative experience, susceptible to individual differences in linguistic skills and developmental history.

A degree of caution is appropriate here though, for two reasons. One is that the amount of data available on deaf individuals with hallucinations is still very meagre: Atkinson et al. (2007), for example, reported on a total sample of 27 individuals, and included some subgroups containing only two people. The other reason, referred to in Inner Speech and Variations in Linguistic Experience, is that very little is known about everyday use of inner speech, inner sign, or any other equivalent in the deaf population. As such, it would be unwise to draw any strong conclusions about “typical” inner speech or AVHs in deafness without knowing more about what is typical in the inner experience of deaf people.

While the greater proportion of research interest has concerned AVHs in people with schizophrenia, inner speech has also been implicated in other forms of adult psychopathology. Rumination is known to be an important feature of depression, and the repetitive concentration on negative thoughts is often described in primarily verbal terms (Nolen-Hoeksema, 2004). In this literature, specific engagement in inner speech has not always been tested, meaning that the verbal nature of rumination has perhaps been more assumed than demonstrated. Nevertheless, some recent studies have highlighted strong verbal and auditory features of depressive states (Moritz et al., 2014; Newby & Moulds, 2012) and drawn specific links between inner speech and depression (Holmes, Lang, & Shah, 2009; Holmes et al., 2006).

For example, Moritz et al. (2014) asked people with mild and moderate depression to report on the sensory phenomenology of their depressive thoughts and ruminations by completing a web-based measure, the Sensory Properties of Depressive Thoughts Questionnaire, which asked about bodily/tactile experiences, visual sensations, and auditory properties, such as experiencing an “inner critic” (p. 1050). Distinct auditory properties were reported by 31% of the sample, which was more common than visual experiences (27%) but less common than bodily experiences (40%). The presence of sensory experiences in depressive thoughts was consistent with a prior, smaller study by Newby and Moulds (2012), although in that case visual experiences were more common than auditory experiences, both for ruminations and intrusive memories. Such studies have been interpreted as showing that verbal depressive thoughts either have their own sensory qualities or are accompanied by concurrent imagery, although investigators do not always ask about the verbality of these cognitions: Newby and Moulds (2012) specifically asked their participants about frequency of verbal thoughts, but Moritz et al. (2014) did not.

More specific evidence of a role for inner speech or verbal thinking in depression has come from studies by Holmes and colleagues. Relative to visual mental imagery, instructions to think verbally about hypothetical scenarios can lead to reductions in mood (Holmes et al., 2006) and susceptibility to a subsequent negative mood induction, even when the imagined scenarios are positive (Holmes et al., 2009). Holmes et al. (2009) have argued that this apparently paradoxical feature of verbal thinking reflects the less immersive qualities of inner speech compared to mental imagery, and the capacity to make unfavorable comparisons when thinking from a more abstract position. Within nonclinical populations, research with elementary schoolchildren has reported associations among self-reported rates of positive self-talk, self-esteem, and depression (Burnett, 1994), while overly evaluative forms of inner speech appear to relate to low self-esteem in university students (Alderson-Day et al., 2014).

Stronger and more specific links between psychopathology and inner speech are evident in research on worry and anxiety. Worry is an example of repetitive thinking that is typically defined as being negative, uncontrollable, and aimed at some form of ill-defined problem-solving, such as a problem with no clear solution (see Watkins, 2008, for a review). Worrying often seems to take a verbal form, and this can have an exacerbating effect in contrast to negative thought in other modalities (such as visual imagery). For instance, Stokes and Hirsch (2010) encouraged a group of self-reported high worriers to engage in either visual imagery or verbal thinking about a worrying topic. Verbal worrying was associated with an increase in negative intrusive thoughts, while visual imagery was associated with a decrease in intrusions (see McCarthy-Jones, Knowles, & Rowse, 2012, for a contrasting example involving hypomania). The tendency for worrying to be linked specifically to verbal processes is consistent with prior research in generalized anxiety disorder (GAD): Behar, Zuellig, and Borkovec (2005) asked a general participant sample and a sample of people with GAD and posttraumatic stress disorder traits to report on their verbal thoughts (“words you are saying to yourself”) and mental imagery (“pictures in your mind”) during recall inductions for worry and trauma. Worry experiences were predominantly verbal in form, while trauma recall was largely imagery-based. Moreover, worrying was more generally associated with a rise in anxious affect during the experiment, while trauma recall showed a closer link to depressive thinking.

As noted in How do Adults Experience Inner Speech?, more evaluative forms of inner speech correlate with higher levels of nonclinical trait anxiety in university students (McCarthy-Jones & Fernyhough, 2011). Research has also linked anxious self-talk with greater anxiety symptoms in children and adolescents (e.g., Kendall & Treadwell, 2007; Sood & Kendall, 2007), although such studies arguably suffer from concerns about content overlap between different self-report measures. As in the example of positively and negatively valenced self talk (Calvete et al., 2005), separating the linguistic phenomenon from the psychopathological state is problematic when both self-report measures ask about similar internal states. For this reason, mood-manipulation studies such as that of Stokes and Hirsch (2010) provide more reliable indicators of the relations between inner speech and anxiety.

## The Neuropsychology of Inner Speech

Before considering how developmental, cognitive, and neuroscientific findings on inner speech might be integrated into a comprehensive model, we briefly consider what neuropsychological research has contributed to the understanding of inner speech. Generally, evidence from this area has largely supported the idea that inner speech plays an important role in adult cognition, while also shedding light on the relationship between overt and covert speech.

Prior to fMRI research on the topic, neuropsychological cases played an important role in establishing the neural basis of verbal working memory. Baddeley and colleagues have argued that the phonological loop system does not require the same neural systems as overt speech production, based in part on evidence that working memory impairment was more closely associated with damage to the supramarginal gyrus in the parietal cortex, and because of double dissociations between samples with speech planning and speech production difficulties (see Baddeley & Logie, 1999, for a review of these arguments). However, subsequent neuroimaging studies on verbal working memory have not always supported this distinction (e.g., Marvel & Desmond, 2010), and neuropsychological studies show examples of both overlap and separation in overt and covert speech processes.

For instance, two studies by Geva and colleagues reported on inner speech and language skills in aphasia (Geva et al., 2011, 2010). In a behavioral study, Geva et al. (2011) tested 27 patients with poststroke aphasia and 27 healthy controls on a range of language tasks, including rhyming tests of inner speech. Compared with controls, patients were impaired on both inner and overt speech, and performance in both was closely correlated. However, there were also individual cases of intact inner speech but not overt speech, or vice versa, suggesting a possible dissociation between the two domains. Geva et al. (2011) used voxel-based lesion mapping to examine the neural correlates of aphasic impairments in 17 of this sample. Impaired performance on inner speech tasks (rhyming and homophone judgment) was observed to correlate with lesions to left pars opercularis (inferior frontal gyrus) and the supramarginal gyrus, relations that remained even when overt speech performance and working memory skills were taken into account. Such data do not prove a dissociation between inner and overt speech, but they do support the notion that inner speech is not simply identical to overt speech processes at a neural level.

Vercueil and Perrone-Bertolotti (2013) reported on a case study of a woman with epilepsy who experienced jargon-like inner speech during seizures. The experience of jargon in overt aphasia is well-documented, but very few accounts exist of jargon in inner speech, most likely due to difficulties in comprehension and reporting of such an experience by patients. In this case study, the patient was able to report on her experience of jargon-like inner speech during seizures:

Her written report mentioned the fact that during her seizures, even inner speech became incomprehensible, with the perception of an inner jargon which remained self-sustained throughout the seizure even though it sounded strange (she literally wrote: “Incomprehension of inner language (thought is unintelligible), and if I try to repeat inner language out loud, incomprehensible words come out (at any rate I don’t understand them!)” (Vercueil & Perronne-Bertolotti, 2013, p. 308).

The authors argue that this provides evidence of shared mechanisms in overt and inner speech, in contrast to the findings of Geva et al. (2011). It is not clear, however, that the two studies are mutually inconsistent, given that areas required for producing overt and inner speech could largely overlap and yet also draw on unique resources. Irrespective of that issue, though, such descriptions highlight the possible separation of monitoring and comprehension skills from production in inner speech.

Levine, Calvanio, and Popovics (1982) described the case of a 54-year-old man who lost his ability to produce language after a mild right hemiparesis, and consequently was unable to generate inner speech, although he did retain an ability to read (with some difficulty). Levine et al. proposed that the patient’s preserved language skills were based on highly developed visual imagery, supported by his general competence on spatial tasks (such as copying complex figures). Another case study of aphasia, in this case following a stroke in language-relevant areas of left temporal lobe, was documented in the autobiography of Dr. Jill Bolte Taylor (Taylor, 2006). Taylor referred to “the dramatic silence that had taken residency inside my head” (pp. 75–76) in describing her loss of inner speech and a range of associated difficulties such as memory retrieval. Morin (2009b) interpreted Taylor’s loss of inner speech as causing an impairment of a sense of individuality and capacity to reflect on the self, consistent with his proposal that inner speech is involved in self-awareness and the creation of a sense of self (Morin, 2005).

Finally, two studies by Baldo and colleagues examined the impact of damage to language regions on problem-solving and reasoning (Baldo, Bunge, Wilson, & Dronkers, 2010; Baldo et al., 2005). Baldo et al. (2005) tested the role of language in supporting task performance on the Wisconsin Card Sorting Test in (a) stroke patients with impaired language abilities, and (b) neurologically intact adults under articulatory suppression conditions. In the clinical group, performance on the WCST was positively correlated with language skill (naming and comprehension), as were matrix reasoning skills. In the nonclinical group, who completed the WCST with and without articulatory suppression, performance was consistently worse when inner speech was blocked, although similar effects were also seen for a visuospatial distractor condition.

In a second study, Baldo, Bunge, Wilson, and Dronkers (2010) examined problem-solving performance on Raven’s Color Progressive Matrices in a sample of 107 patients with left hemisphere stroke lesions and varying levels of language impairment. Stroke patients with aphasia were significantly worse in their problem-solving than were patients without aphasia, particularly for puzzles requiring relational reasoning rather than visuospatial matching. Furthermore, impaired performance in relational reasoning puzzles was related to lesions to the left middle and superior temporal areas of the cortex. Taken together, these studies suggest that damage to typical language areas could impede performance during certain kinds of problem-solving, even when the task does not clearly require language to be attempted.

## Toward an Integrated Cognitive Science of Inner Speech

As the foregoing review has demonstrated, a growth of research interest in inner speech has coincided with methodological progress in techniques for eliciting and manipulating it experimentally and imaging its neural substrates (Fernyhough, 2013). At the same time, empirical advances have not always been tightly linked to theoretical issues concerning the development, phenomenology, and possible cognitive functions of inner speech. In this section, we consider outstanding challenges and obstacles remaining for an integrated cognitive science of inner speech, beginning with the question of whether inner speech represents a unitary process that can be adapted to the demands of different tasks and contexts.

### Toward a Unifying Account of Inner Speech

**Figure 1 fig1:**
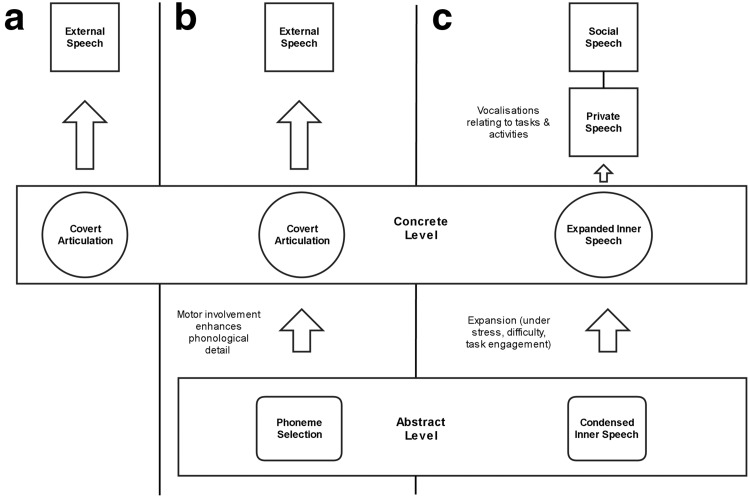
Inner speech (a) as covert articulation, (b) as a flexible abstraction, and (c) in condensed/expanded forms.

We begin by considering whether the findings reviewed above fit with what might be termed a “minimal” account of inner speech. A number of studies still primarily associate inner speech with a unitary process equivalent to covert articulation (Figure 1a), with specific functions in maintenance of verbal information and covert planning of speech acts (Geva et al., 2011; Marvel & Desmond, 2010; Scott, 2013). This view of inner speech is reflected in the selection of tasks in neuroimaging studies, in which participants are typically asked to repeat words or sentences, or judge the stress of specific syllables. The research reviewed here, however, has implicated inner speech in a variety of cognitive processes including social cognition, executive function, and imagination, with functional properties of inner speech changing considerably with age and linguistic experience. There is also evidence, from psycholinguistic and phenomenological studies, to suggest that inner speech can vary in its phonological, semantic, and syntactic properties, from abstract to concrete, from condensed to expanded, and from inner speaking to inner hearing. A minimal view of a single form of inner speech deployed for such varied functions in such different contexts, and with such differing phenomenology, would at the very least require specification of how a unitary process could operate.[Fig-anchor fig1]

Oppenheim and Dell’s (2010) flexible abstraction hypothesis is an example of an account in which a single underlying process can be adapted for differing task demands. In their model, inner speech is primarily an abstract verbal representation at the level of phonemic selection, whose degree of featural specification can be adjusted depending on the degree of articulation deployed (see Figure 1b). The contrast between condensed and expanded inner speech in Fernyhough’s (2004) model could be viewed in a similar way, although in that case what varies between condensed and expanded forms is the semantic and syntactic complexity of the inner speech representation, as well as its phonological detail (see Figure 1c).

**Figure 2 fig2:**
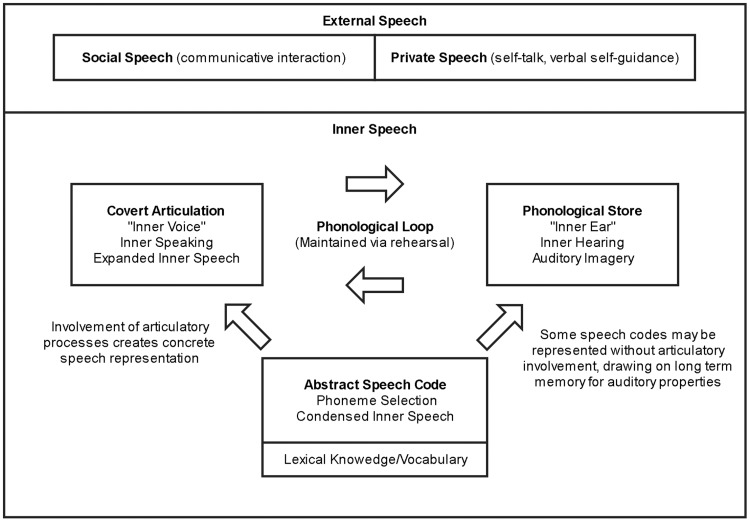
A multicomponent model of inner speech, incorporating developmental, working memory, and psycholinguistic features.

If the core of inner speech is considered as an abstract code containing a combination of semantic, syntactic, and phonological information, one way to account for its apparent varieties is to think about that “kernel” or abstract code being unpacked in different ways, depending on the recruitment of additional cognitive resources. An “utterance” in inner speech could be articulated to a greater or lesser degree, depending on the relative deployment of speech motor processes. In the case of greater articulatory involvement, inner speech would resemble something akin to an “inner voice,” which would usually correspond to the speaker’s own. According to working memory models, continued rehearsal of the inner speech utterance via the phonological loop would keep the trace maintained in an “inner ear” (see Figure 2).[Fig-anchor fig2]

For much of the time this may be all there is to inner speech: a relatively abstract speech code that can be more or less featurally specified, reverberating between articulatory and phonological store components of a verbal working memory system. However, if reports of “inner hearing” are also considered variations in inner speech experience, then articulation may not be the only way of unpacking such representations. Recruitment of phonological associations from memory, without articulation, could give rise to inner hearing experiences, or inner speech that involves the experience of other people’s voices. Specifically trying to produce another’s voice in inner speech (or, as some would term it, auditory imagery) would draw even more upon memory for phonological information to fill out the auditory detail of the trace. Based on the neuroimaging findings discussed in Inner Speech in the Brain, the relative involvement of articulatory and phonological information in this process will correspond to the use of inferior frontal areas (Broca’s area, insula) and posterior temporal structures (superior and middle temporal gyri, temporoparietal junction), respectively.

As an offline, abstract code, inner speech can act as a representational tool, for internal planning, rehearsal, or rumination, or for filling in the gaps in the absence of relevant information (e.g., Miyake, Emerson, Padilla, & Ahn, 2004). The presence or absence of a task could determine the structure of inner speech deployed in a particular situation. Conditions where a given response needs to be maintained or regulated (as in set-shifting) may be more likely to require an expanded and task-specific form of inner speech (and in some cases private speech), while more exploratory, open-ended forms of verbal thinking could remain at a more abstracted, condensed level.

This view of inner speech as a multicomponent system points to the value of taking a developmental perspective on this complex and varied experience. Fernyhough (2010) has proposed that inner speech can be considered as resulting from the development of a functional system (Luria, 1965; Vygotsky, 1934/1987). Luria construed the executive functions as a functional system involving the interaction of hierarchically organized subsystems with diverse neurological foci (Luria, 1965). Rather than seeking the cause of executive functioning development solely in brain maturation, Luria held that that social interaction shapes emerging cortical organization in the preschool years: “Social history ties those knots which form definite cortical zones in new relations with each other, and if the use of language . . . evokes new functional relations . . ., then this is a product of historical development, depending on ‘extracerebral ties’ and new ‘functional organs’ formed in the cortex” (Luria, 1965, p. 391). In Luria’s view, a new form of executive functioning emerges when prelinguistic capacities for monitoring, planning, and inhibition of behavior enter into interfunctional relations with language abilities (Fernyhough, 2010). This corresponds to Vygotsky’s “revolution” in development, when preintellectual language and prelinguistic cognition become fused (Vygotsky, 1930–1935/1978) in the emergence of self-regulatory private speech and then inner speech.

In this view, inner speech represents a functional system whereby initially independent neural systems are “wired together” in new ways by social experience. The basic tools necessary for this developmental progression—such as a phonological loop and the capacity for verbal rehearsal—may already be in place relatively early in childhood, serving core functions of speech production and language learning. Subsequently, the effects of social interaction and communication shape how those tools are put to use in supporting cognition from middle childhood onward. By adolescence and adulthood, changing patterns of deployment of the components of the functional system link nominally separate systems of executive skill, but not necessarily in the same way as before.

Another example of the development of a functional system is the emergence of theory of mind (ToM). ToM capacities have been proposed to result from early forms of intentional-agent understanding becoming modulated by language (Fernyhough, 2008, 2010)—accounting for, *inter alia*, evidence for very strong relations between ToM reasoning and language in childhood, and effects of inner speech disruption on ToM performance in adulthood (Newton & de Villiers, 2007). One implication of this view is that the functional system(s) of ToM will evidence shifting patterns of relation across age of the component neural systems, consistent with evidence that ToM networks in the brain “crystallize” from more diffuse agglomerations of neural foci in the course of childhood (Saxe, Carey, & Kanwisher, 2004).

A functional systems approach thus entails shifting relations among constituent neural systems over the course of development which will not necessarily represent their eventual pattern of interaction in adulthood (Fernyhough, 2010). In the case of inner speech, early interrelations among language and other systems will likely change as the child develops, perhaps incorporating ToM-relevant regions that are different from those identified in adulthood. Charting these emerging dynamic relations is a challenge for future research. One possibility is that the generation of overt self-regulatory private speech gradually captures the emerging ToM system, or indeed other neural systems, so that the child is able to represent a perspective on her own self-generated speech. In the case of ASD, a delay or deviation in the emergence of ToM, perhaps at the level of very early intentional-agent understanding, may prevent the yoking of language and ToM systems necessary for internal dialogue.

A functional systems approach also has relevance for understanding data from neuropsychological studies of inner speech. Particularly interesting in this respect is the case of acquired aphasia, whose influence on ToM and executive functioning abilities has been the subject of several recent studies (e.g., Willems, Benn, Hagoort, Toni, & Varley, 2011). If language is essential for the development of dialogic inner speech, then individuals with acquired aphasia might be expected to be at a disadvantage in tasks requiring inner dialogue. However, typical language development prior to the onset of aphasia may allow the development of dialogic inner speech in childhood and adolescence, creating the cognitive structures necessary for dialogic thinking even if one of the component systems is subsequently damaged and another neural system has to be recruited to replace it. Central to Luria’s reasoning about functional systems was the idea that brain lesions will have differing significances depending on where they occur within an emerging functional system and at what point in development (Luria, 1965; Vygotsky, 1934/1987).

**Figure 3 fig3:**
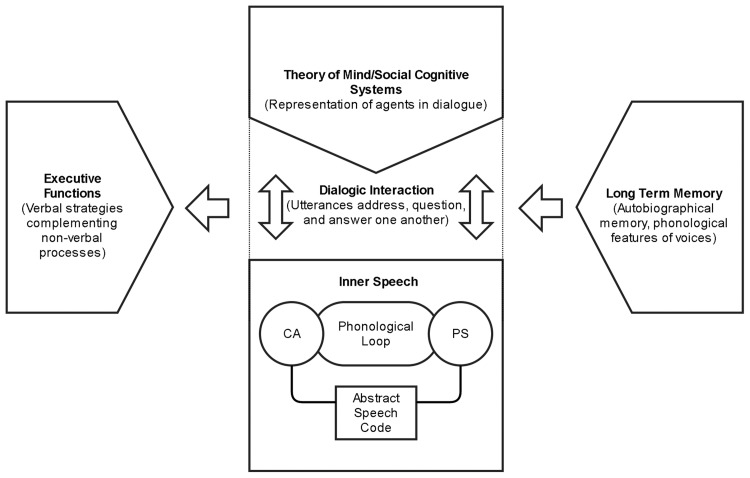
The inner speech system and its interaction with executive functions, theory-of-mind, and long-term memory. (CA = covert articulation; PS = phonological store)

Adopting a developmental approach thus points to further developments in how inner speech can be conceptualized and modeled. On this view, inner speech will be shaped by the individual’s linguistic and social experiences, possessing the qualities of being evaluative, discursive, or addressed to others, because it retains some of the pragmatic characteristics of external communication. We have also noted that developmental considerations motivate the drawing of distinctions between monologic and dialogic inner speech (Fernyhough, 1996), a distinction that has been supported by data on self-reported experiences of inner speech (Alderson-Day et al., 2014; McCarthy-Jones & Fernyhough, 2011). The dialogic quality of some forms of inner speech is plausibly supported by the recruitment of ToM systems as described above. Figure 3 represents a model incorporating the inner speech model depicted in Figure 2, with the addition of the social–cognitive processes that may underlie inner dialogue. Fernyhough has proposed that the dialogicality of inner speech can be interpreted as the cognitive provision of an “open slot” (Fernyhough, 1996, 2009a) within which a linguistically manifested perspective generated in the inner speech network is represented while an answering perspective is generated. Alongside this, monologic or dialogic forms of inner speech can be deployed to support nonverbal executive processes where this is required (as in the examples of switch tasks, or cognitive control). Representation of voices and situations will also require retrieval of autobiographical information from long-term memory, as in the case of replaying a particular conversation in the mind.[Fig-anchor fig3]

### Implications of a Multicomponent Account of Inner Speech

One implication of a multicomponent view of inner speech is that everyday instances of the phenomenon are likely to be richer and more complex than conceptualizations of inner speech in typical laboratory studies, which have mostly drawn on a Watsonian view of verbal thinking as overt speech without articulation. Two recent neuroimaging studies have begun the attempt to address this issue: the first by using experience sampling (Kühn, Hurlburt, Alderson-Day, & Fernyhough, 2014) and the latter by evoking dialogic inner speech (Alderson-Day et al., 2015).

A perennial problem for neuroimaging research has been how to tie data on neural activations in the scanner to subjective assessments of experience (Fell, 2013). This problem is particularly acute in the study of inner speech, where experimental manipulations intended to elicit inner speech may result in experiences quite dissimilar to ordinary spontaneous inner speech (cf. Jones & Fernyhough, 2007, and Hubbard, 2010). Silent reading has been used as a paradigm for studying featural properties of inner speech (Yao, Belin, & Scheepers, 2011), but even this is by no means certain of tapping spontaneous examples of verbal thinking (Fernyhough, 2013). In an attempt to bridge this gap, Kühn, Hurlburt, Alderson-Day, and Fernyhough (2014) combined the DES method with fMRI to examine randomly beeped moments of inner experience while participants took part in resting-state scans. Participants were trained in the DES method for 1 week before completing a week of MRI scans that contained random DES beeps. Reporting on a case study of one participant who self-described as regularly experiencing inner speech, Kühn et al. observed consistent activation of left inferior frontal gyrus for beeps associated with verbal thinking in general, and inner speech in particular. There was also some preliminary evidence for localized distinctions between experiences of inner speaking and inner hearing. Conclusions are necessarily limited by the single-case design, but such findings at least act as a proof of principle that spontaneous inner speech can be studied in depth both qualitatively and neurally.

Alderson-Day et al. (2015), in contrast, examined the neural basis of dialogue-like verbal thinking. When participants were asked to generate dialogue in their heads, in contrast to matched monologic scenarios (for instance, telephoning a relative and having a conversation, as compared with leaving a voicemail), a widespread bilateral neural network was implicated, including medial frontal regions, precuneus, posterior cingulate, and right posterior superior temporal gyrus. Activation in the latter region also significantly overlapped with activation linked to ToM reasoning (Alderson-Day et al., 2015). These findings were interpreted in terms of dialogic inner speech involving an interaction between language and social cognition networks. The findings suggest a neural instantiation of this interaction between a system for generating an element of inner speech and a system for responding to it from the perspective of another person—in other words, for the provision of an “open slot” within which an utterance generated in the inner speech network is represented while a dialogic response is being generated.

In addition to providing theoretical detail on the cognitive and neural instantiations of dialogic inner speech, Alderson-Day et al.’s study responds to the challenge set by Jones and Fernyhough (2007) to develop more ecologically valid methods for eliciting inner speech. A further phenomenological feature that is worthy of continued empirical study is the distinction, derived from Vygotsky’s theory, between condensed and expanded inner speech (Fernyhough, 2004). As noted, this distinction bears strongly on the debate about how much inner speech retains phenomenological features of overt speech, such as tone, accent, and timbre (see What is the Relation Between Inner Speech and Overt Speech?). Rather than specifying levels of featural richness for all inner speech, Fernyhough’s (2004) model proposes flexible movement between condensed and expanded forms in typical spontaneous inner speech, an idea that receives some support from studies involving the VISQ self-report instrument (Alderson-Day et al., 2014; McCarthy-Jones & Fernyhough, 2011).

Although it has not yet been the focus of neuroimaging studies (not least because of the difficulty, noted above, of capturing heterogeneous forms of inner speech in the scanner), it is possible to speculate on the neural substrates of condensed and expanded inner speech. Because it is not phenomenologically full-blown, condensed inner speech could be predicted not to activate areas involved in detailed phonological representation, such as the STG. The “pure meanings” of condensed inner speech, instead, may be expected to be based in areas associated with semantic representations and abstract knowledge about semantic categories. The ventral posterior middle temporal gyrus has been proposed to provide a “lexical interface” bringing together semantic and phonological representations (Hickok & Poeppel, 2007, p. 395), while the anterior temporal pole has been linked to modality-invariant, abstract representations of semantic categories (Visser, Jefferies, & Lambon Ralph, 2010). It is possible that the move from condensed to expanded inner speech will involve translation of such representations into something more fully voiced, via articulation in the left IFG and phonological representation in STG structures.

### What are the Relations Among Inner Speech, Inner Hearing, and Auditory Imagery?

Another phenomenological distinction that has emerged from studies of the subjective experience of inner speech is the distinction between inner speaking and inner hearing. This distinction stems from Hurlburt’s (e.g., Hurlburt et al., 2013) work showing that some DES participants distinguish episodes in which they feel themselves to be the producers of the speech from those in which inner speech is more passively received (as in listening to one’s own voice on a recording). Such a distinction is absent from many areas of inner speech research, including work on child development and studies of adult executive function. In contrast, evidence for separable mechanisms for inner speaking and hearing is provided in the literature on verbal working memory and auditory imagery: as noted above, the “inner voice” and “inner ear” can both be disrupted under conditions of articulatory suppression and purely auditory interference, but can show separable interference effects depending on the kind of task deployed (see Smith, Reisberg, & Wilson, 1992, for a review). Indeed, the “articulatory loop” in working memory was renamed the “phonological loop” by Baddeley and colleagues precisely because of evidence that phonological information could be retained in working memory even when articulation is blocked (Baddeley & Larsen, 2007).

What Hurlburt’s observations add is the suggestion that, at least for some people, the everyday experience of “inner speech” may not always involve an experience of actively speaking. Inner speech may be generated in one’s own voice or in that of another, but the experience of it—in the sense of an internal representation of verbalized language—will not necessary feel as though one is involved in its production. If correct, this raises important questions for developmental accounts of inner speech, such as what components underlie inner speaking and inner hearing, and when they are in place. There may also be implications for theories of psychopathology: would a person who reports more frequent experiences of inner hearing than of inner speaking be more prone to unusual experiences, such as hallucinations or passivity phenomena?

It has also been suggested that inner speech is a special case of a more general phenomenon of auditory imagery. For example, Levine et al. (1982) defined inner speech as the “subjective phenomenon of talking to oneself, of developing an auditory-articulatory image of speech without uttering a sound” (p. 391). More recently, Hubbard (2010) reviewed empirical research on auditory imagery and treated inner speech as a form of imagery. On such a reading, inner speech refers to a subset of auditory imagery experiences; namely, just those that happen to include the representation of speech.

In support of such an idea, inner speech and auditory imagery appear to share many similar properties; indeed, some studies using inner speech paradigms refer to it as articulatory imagery or speech imagery. Both inner speech and auditory imagery show evidence of interference under articulatory suppression, for example. Both are also associated with activation in a set of common regions, including inferior frontal gyrus, insula, SMA, and posterior superior temporal gyri (among other regions) in neuroimaging studies (Hubbard, 2010; Price, 2012). Considering inner speech as an example of auditory imagery offers one way of subsuming inner speech and related phenomena into a single class of cognitive processes. One reason for not doing so would be if inner speech appeared to rely on underlying mechanisms or have effects that made it function in a different way to imagined sound.

We argue that there are good reasons to retain the label of inner speech as a related but broadly separable process to auditory imagery. First, although motor processes can affect certain kinds of auditory imagery (Hubbard, 2013), subsuming inner speech within imagery would appear to underestimate its articulatory component, in which words are usually actively voiced and expressed rather than simply being “sounded out.” It is not at all clear—and would seem counterintuitive to suggest—that inner speech is “imagined” in the same way that one can imagine the sound of a siren, or even imagine hearing one’s own voice on a recording, notwithstanding the fact that some individuals may experience inner speech more as a “hearing” than as a “speaking” phenomenon.

In neuroimaging studies, this articulatory involvement is reflected in the general pattern of regions associated with inner speech and auditory imagery. Despite some overlap in activations, inner speech paradigms are commonly associated with left inferior frontal gyrus, left insula, and left STG activation (Fegen et al., 2015; McGuire et al., 1996); in contrast, auditory imagery for speech (whether imagining hearing one’s own voice or another’s) and auditory imagery for other sounds is associated with activation of SMA, posterior parietal cortex, and STG/MTG bilaterally (Zatorre & Halpern, 2005). Contemporary models of speech processing suggest at least two cortical streams affecting speech perception: a left lateralized dorsal stream, connecting speech motor processing (left inferior frontal gyrus and insula) with posterior temporal regions, and a bilateral ventral stream linking hippocampal structures and the inferior and middle temporal gyri (Hickok & Poeppel, 2007). Evidence from Tian and Poeppel (2010), for example, suggests that these separate streams produce differential and contrasting repetition priming effects on speech perception. As such, it seems useful to consider articulated language representations as related but importantly different entities to auditory images more generally.

Second, considering inner speech as a kind of imagery would not seem to fit comfortably with the range of evidence reviewed above. Inner speech is used as planner, regulator, reminder, and commentator across many different contexts, and in some cases would appear to have differential effects to engagement in mental imagery (e.g., Stokes & Hirsch, 2010). Speech representations are arguably unique in their capacity to generate and maintain propositional content while ordinary perceptual processes are still ongoing. Of other modalities, only visual imagery has similar propositional capacity—I can say “the cat is on the mat” or I can create an image depicting that scenario—but images of situations or states of affairs are difficult to generate while visual processing of the outside world is ongoing (e.g., Borst, Niven, & Logie, 2012). In this way, inner speech offers an abstract and flexible code to support ongoing cognitive operations. Perhaps for this reason, inner speech is used much more often as a synonym for thinking that it is for imagery, although usages of both of the latter terms are so broad that their explanatory value is easily questioned. In some cases a distinction between inner speech and imagery has also been framed in terms of the opposition between speaking in one’s own voice and imagining someone else’s voice (Shergill et al., 2001). This, however, would appear to confuse two separable dimensions: first, the extent to which an inner verbal representation is experienced as being articulated rather than being heard, and second, the extent to which a verbal representation has an identity belonging to self or other.

Instead, we advocate an alternative approach utilizing the model depicted in Figure 2 and incorporated into Figure 3. On this view, inner speech and auditory imagery systems overlap in their use of phonological information from long-term memory, but at its core inner speech is an abstract linguistic code, that shares more resources with overt speech production than does auditory imagery. Often, this will involve concurrent deployment of articulatory processes and phonological representations via the phonological loop, such that inner speech has a sensory-motor and auditory phenomenology of its own. In some circumstances condensed or abstracted inner speech may even be unpacked as an inner hearing experience, if no articulation is involved in its expansion.

Although this work has not yet been conducted, the cognitive and neural dimensions of the distinction between speaking and hearing could be assessed by incorporating items into self-report instruments such as the VISQ, and by attempting to capture such experiences spontaneously during neuroimaging (Kühn et al., 2014). As with the suggestion above concerning experience-capture of dialogic inner speech in the scanner, use of a method such as DES to report on spontaneous occurrences of inner hearing could be correlated with ongoing brain activations in a way that would reveal the neural bases of the distinction.

### What are the Relations Between Inner Speech and Mind-Wandering?

Experientially, much of everyday or spontaneous inner speech may also be thought to be similar to verbally based mind-wandering. The growth of interest in cognition in the resting state (Andrews-Hanna, Reidler, Huang, & Buckner, 2010; Buckner, Andrews-Hanna, & Schacter, 2008) has recently been accompanied by a more specific interest in the particular modalities present in mind-wandering or stimulus-independent thought (Delamillieure et al., 2010; Doucet et al., 2012; Gorgolewski et al., 2014). From the results of a semistructured questionnaire assessing subjective experience during fMRI, Delamillieure et al. (2010) reported that 17% of resting-state experiences described by their participants were language-based. It has been suggested by Perrone-Bertolotti et al. (2014) that verbal mind-wandering may involve an abstract form of inner speech while voluntary verbal thought may have a more concrete form, and that this distinction might map on to the anticorrelation between default mode network activation and task-positive activation of language networks. Although there is some preliminary evidence in support of such an idea (Doucet et al., 2012), no studies to date have captured specifically verbal mind-wandering in action, and much of mind-wandering may also involve internal representation of other kinds, such as visual imagery. As such, the incidence of inner speech in the resting state remains largely unclear.

Nevertheless, the idea of concrete and abstract inner speech mapping on to voluntary inner speech and involuntary mind-wandering is an intriguing one, with potential overlaps with some other concepts described above, such as the distinction between condensed and expanded forms of inner speech. Fernyhough’s (2004) model would predict that resting-state inner speech would be predominantly condensed, as the theory holds that reexpansion happens when cognitive challenge increases. If there is no task, there is by definition no cognitive challenge, and thus the default mode of condensed inner speech would predominate.

Challenges for future research include developing improved methods of assessing subjective experience in the scanner that will allow a closer integration of mind-wandering phenomenology with information on neural activations. The methodology described by Kühn et al. (2014) suggests one possible approach to studying verbal mind-wandering using an experience sampling design. Given the proposed role for inner speech in resting-state cognition, there is also a need for functional connectivity studies focusing on how the inner speech network modulates the activities of the default mode network and various task-positive networks, in both healthy participants and patients with disorders such as schizophrenia.

### Inner Speech and the Forward Model in Auditory Verbal Hallucinations

As previously noted, perhaps the most prominent use of inner speech as an explanatory concept is in the domain of auditory verbal hallucinations (AVHs). Fernyhough (Fernyhough, 2004; Fernyhough & McCarthy-Jones, 2013) has argued that attention to the multifaceted nature of inner speech, particularly the distinction between its condensed and expanded forms, can be instructive in accounting for the paradoxical “alien yet self” quality of such experiences (Leudar & Thomas, 2000). What the foregoing review highlights, in demonstrating the heterogeneity and complexity of these processes, is that disruptions to inner speech (resulting from or leading to psychopathology) are likely to have equally varied effects.

As reviewed in Adult Psychopathology, prominent models of AVH posit that voices arise from a failure of self-monitoring, whereby internal speech productions are misattributed to external sources. Hallucinations are posited to arise from a disruption to signals sent between areas responsible for speech production and perception (e.g., Broca’s area and Wernicke’s area). One criticism of such an explanation is that the voices heard in AVH do not resemble the person hearing them: they both feel alien and resemble the voices of other people, and say things that the hearer may not normally say. However, if inner speech is taken to be the “raw material” of AVH, then this will potentially involve many different kinds of speech representation, varying in phonological detail and identity depending on the personal experience of that individual. And if inner speech is a multicomponent phenomenon, then multiple resources will be recruited to represent more or less featurally rich inner speech, or inner speech in the voices of other people, meaning that many more pathways than a “typical” Broca–Wernicke network may be involved. Potential pathways suggested by MRI studies of hallucinations include right hemisphere homologues of language areas (Sommer et al., 2008), hippocampal cortex (Diederen, Neggers et al., 2010), and subcortical structures (Hoffman, Fernandez, Pittman, & Hampson, 2011).

Evidence of social-cognitive involvement in dialogic inner speech raises intriguing questions as to the role of ToM in representing inner voices. If ToM is drawn upon to shape representations in inner speech, it could be that mental states, rather than verbal representations per se, are being misattributed in the case of AVH (Bell, 2013; Wilkinson & Bell, in press); that is, the input of ToM processes into internal monologue or dialogue could in themselves be disrupted or atypical (e.g., Koster-Hale & Saxe, 2013). Further research on the interrelation of involuntary inner speech and verbal mind-wandering may also shed light on this question, as they have implications for the sense of agency and ownership conferred on one’s own inner speech. Where this is disrupted, inner speech may feel like it is coming from another agent or entity, as is the case in examples of thought insertion (Langland-Hassan, 2008).

A remaining conceptual question for such self-monitoring accounts of AVH is what part of this system should be equated to the experience of inner speech. Due to their basis in motor theory, such accounts typically posit that hallucinations arise from a mismatch in the comparison between an action and a forward model of its predicted sensory consequences: In the case of AVH, a mismatch between an episode of inner speech and its predicted state gives rise to an anomalous internal representation of speech. However, in contrast to overt speech, inner speech has no sensory consequences of its own by definition, leaving its position in self-monitoring accounts unclear.

One solution is to posit that, because inner speech uses many of the same speech-motor processes as overt speech, it is still accompanied by the issuing of a forward model. That is, if someone engages in inner speech (or, effectively, subvocal speech) but does not realise it, prediction signals will still be sent to sensory areas to create an experience of a voice. In contrast, several recent authors have recently proposed that the normal experience of inner speech in some way equates to either the forward model itself, or to the sensory outcomes it predicts. Scott (2013) presented evidence that generation of inner speech attenuates the perception of external sounds, consistent with the view that a sensory prediction of the utterance in question interferes with the perception of an external sound. In two experiments, Scott, Yeung, Gick, and Werker (2013) showed that generating inner speech “captured” the perception of ambiguous auditory sounds, suggesting the functioning of a forward model in shaping the perception of an incoming sensation, both when the vocalization was generated in inner speech and when it was silently mouthed (see Hubbard & Stoeckig, 1988, for a similar example of priming effects during auditory imagery).

Elsewhere, Pickering and Garrod (2013) have proposed that inner speech might be a stripped-down product of forward models that enables the detection of errors in overt speech before they occur, while Oppenheim (2013) has suggested that inner speech may constitute an internal loop consisting entirely of forward model predictions, bypassing the need for recruitment of standard production and comprehension systems. When one considers Oppenheim and Dell’s (2010) findings of greater phonological detail in inner speech being associated with greater articulatory involvement, this would seem to fit with a conception of inner speech reflecting a predicted state with a level of featural detail that varies according to the degree of articulatory motor involvement.

Specifying the role of the predicted state in inner speech production is an important challenge for future research on the relations between everyday inner speech and atypical experiences such as AVH. In one respect, an account of inner speech as attenuated action is congruent with the Vygotskian view that it represents an internalized (and thus truncated) version of external social exchanges. Particular challenges include accounting for the varied phenomenology of inner speech (particularly the processes such as abbreviation and condensation that are proposed to accompany internalization), and explaining how inner speech has cognitive efficacy in domains such as the self-regulation of cognition and behavior, if indeed it is considered to have its basis in internal speech predictions. Finally, a further problem is that self-monitoring theories in general have been criticized for failings in accounting for the evidence from psychopathology, including the high variability of positive symptoms among patients (Frith, 2012).

### Do We Really Need Inner Speech?

A final question, again prompted by phenomenological investigation of inner speech, is whether we overestimate its presence and relevance. As Hurlburt et al. (2013) note, presuppositions about the ubiquity of inner speech may limit the accuracy of efforts to report on its incidence. Introspective methods such as DES tend to result in lower incidence ratings than self-report measures. Alderson-Day and Fernyhough (2014) argue that DES may underestimate the incidence of inner speech for various reasons, including that the DES method may not be sensitive to transformations such as condensation (although see Hurlburt & Heavey, 2015). There may be further, more profound reasons why differing assessments of inner experience can lead to such divergent characterizations of the phenomena. Hurlburt and Heavey (2015) argue that instruments such as the VISQ offer at best a self-theoretical description of any one participant’s inner experience. Based on their observations of participants’ first DES sessions, they propose that (at least until participants become appropriately skilled through engagement in an iterative process like DES) people are frequently misguided about their own experience (Hurlburt et al., 2013). Although it seems counterintuitive to suggest that individuals can be wrong about their own experience (cf. Jack, 2004), the question of how training in reporting on one’s own inner experience might increase the accuracy of self-reports of inner speech remains an intriguing one for future research.

Whether or not Hurlburt is correct, inner speech would certainly appear important to many people’s subjective views of their own experience. Evidence from bilingualism points to inner speech in first and second languages being associated strongly with personal identity and history (de Guerrero, 2005). Correspondingly, loss of inner speech following brain injury, perhaps through its influence on the self-narration that typically accompanies everyday experience, may lead to the diminution of a sense of self (Morin, 2009b). Evidence from cognitive studies also points to a prominent role for inner speech in a diverse range of functions, particularly in childhood. In adulthood, the cognitive benefit of verbalized strategies may wane or be superceded but, for many individuals, the importance of inner speech as a private activity at the core of experience would seem to remain.

### Further Conceptual Issues

Researchers who have approached inner speech from a Vygotskian perspective have observed that such an approach can be valuable in accounting for the phenomenological richness and diversity of inner speech, along with its multifunctional properties. Several conceptual issues need to be resolved, however, before the value of the Vygotskian position can be fully assessed. One requirement is a more detailed specification of the important concept of internalization (Fernyhough, 2008), where further progress is needed in characterizing the transition of socially configured functions from the interpsychological to the intrapsychological planes (Fernyhough, 2009a), along with the cortical reorganizations proposed by Vygotsky and Luria to accompany that process (Fernyhough, 2010). In the case of inner speech, this problem translates into an issue of specifying the cognitive and neural processes underlying the transformations such as abbreviation proposed by Vygotsky.

Finally, it needs to be considered whether a richer account of inner speech, as outlined here, entails a claim for the constitutive involvement of language in thinking (e.g., Carruthers, 2002). Such a claim does not necessarily follow. The present characterization of inner speech may be more appropriately conceived as a model of how typically developing humans perform some forms of high-level cognition, without meaning that such processes necessarily require inner speech. Given the progress that remains to be made in studying this form of speech scientifically, any claim that this involvement of language is constitutive would be premature. In addition, we would hold that claims about the role of inner speech as a “language of thought” are fraught with difficulty (Machery, 2005), through being largely untestable and often conceptually muddled.

Also remaining is the question of what, at root, inner speech is *for.* Adopting an evolutionary perspective, Agnati et al. (2012) have considered inner speech as an exaptation, in Gould and Vrba’s (1982) sense of a feature that has become diverted from its initial evolutionary “purpose” (see also Oppenheim, 2013). On Agnati et al.’s view, inner speech initially developed as a positive tool for planning and internal dialogue. AVHs and other putative pathologies of inner speech such as rumination could be considered as “mis-exaptations,” defined as adaptations that have reached a degree of specialization that is deleterious to the organism. While the focus of Agnati et al.’s account is on some of the psychopathological consequences of exaptation, such an idea may be useful for thinking about inner speech more generally. To go further than Agnati et al., the initial “purpose” of inner speech may not have related to general cognitive planning, so much as to supporting overt speech processing, by enabling internal phonological representation and planning of speech acts. Its exaptation, however, could have come in the application of inner speech to the range of other cognitive domains reviewed above, sometimes in clearly beneficial ways (such as thinking about the future, or regulating behavior), but sometimes in ways that are deleterious to other cognitive functions (such as during pathological worrying). In this sense, much of what we know of inner speech could illustrate its significance as an exaptation: as a motor-based linguistic tool that has by chance created an inner life.

## Conclusions

Inner speech is a paradoxical phenomenon. It is an experience that is central to many people’s everyday lives, and yet it presents considerable challenges to any effort to study it scientifically. Nevertheless, a wide range of methodologies and approaches have combined to shed light on the subjective experience of inner speech and its cognitive and neural underpinnings. In childhood, there is evidence for a central role for inner speech in regulating behavior and supporting complex cognitive functions. In adulthood, inner speech is implicated in many cognitive processes, but there appears to be wide interindividual variation in how inner speech is put to use, both cognitively and experientially. Furthering our knowledge of the range of ways in which inner speech can operate is a research priority, not just for its implications for understanding development, cognition, and psychopathology, but for drawing us toward a richer understanding of human beings’ inner lives.
